# Carbon dioxide adsorption based on porous materials

**DOI:** 10.1039/d0ra10902a

**Published:** 2021-03-31

**Authors:** M. Sai Bhargava Reddy, Deepalekshmi Ponnamma, Kishor Kumar Sadasivuni, Bijandra Kumar, Aboubakr M. Abdullah

**Affiliations:** Center for Nanoscience and Technology, Institute of Science and Technology, Jawaharlal Nehru Technological University Hyderabad Telangana State 500085 India; Center for Advanced Materials, Qatar University P. O. Box 2713 Doha Qatar kishorkumars@qu.edu.qa; Department of Mathematics, Computer Science and Engineering Technology, Elizabeth City State University Elizabeth City NC 27909 USA

## Abstract

Global warming due to the high concentration of anthropogenic CO_2_ in the atmosphere is considered one of the world's leading challenges in the 21^st^ century as it leads to severe consequences such as climate change, extreme weather events, ocean warming, sea-level rise, declining Arctic sea ice, and the acidification of oceans. This encouraged advancing technologies that sequester carbon dioxide from the atmosphere or capture those emitted before entering the carbon cycle. Recently, CO_2_ capture, utilizing porous materials was established as a very favorable route, which has drawn extreme interest from scientists and engineers due to their advantages over the absorption approach. In this review, we summarize developments in porous adsorbents for CO_2_ capture with emphasis on recent studies. Highly efficient porous adsorption materials including metal–organic frameworks (MOFs), zeolites, mesoporous silica, clay, porous carbons, porous organic polymers (POP), and metal oxides (MO) are discussed. Besides, advanced strategies employed to increase the performance of CO_2_ adsorption capacity to overcome their drawbacks have been discoursed.

## Abstract 2

Global warming is considered one of the world's leading challenges in the 21st century as it causes severe concerns such as climate change, extreme weather events, ocean warming, sea-level rise, declining Arctic sea ice, and acidification of oceans.

## Graphical abstract

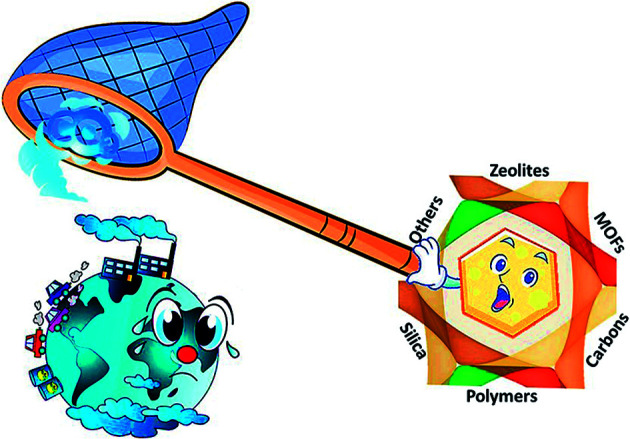

## 1. Introduction

**Fig. 1:**
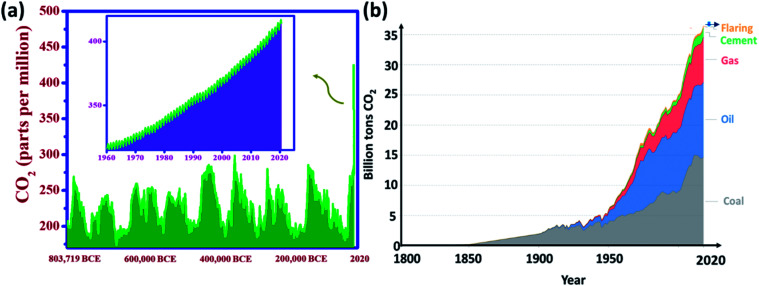
(a) Global atmospheric CO_2_ concentrations over 800 000 years (data taken from ref. 8), (b) global CO_2_ emissions over a period by fuel type (Hannah Ritchie and Max Roser, 2017 (ref. 8)) (Creative Commons BY license).

In recent decades, global warming has become an international issue, and the global average temperature has increased by around 1 °C which is credited to the rise in greenhouse gas emissions. This connection between greenhouse gas concentrations and global temperatures – particularly carbon dioxide emissions – has been practiced throughout the Earth's history.^1,2^ Shifting climate results in extreme weather events (such as storms, droughts, floods, and heatwaves), and a series of potential ecological, physical, and health impacts, altered crop growth, sea-level rise, and disrupted water systems. The 5^th^ Intergovernmental Panel on Climate Change (IPCC) report summarizes the potential impacts of climate change.^3^ For the first time in over 800 000 years, the atmospheric concentration of CO_2_ has not only risen above 300 ppm but also currently, it is well over 400 ppm^4^ (Fig. 1(a)). It is predicted to surpass 500 ppm in 2050 as the energy demand of the world mounts and high levels of coal, oil, and natural gas are consumed.

Fig. 1(b) shows fuel-wise global CO_2_ emissions over a period of 220 years. These emissions rose from 2 billion tons of CO_2_ in 1900 to over 36 billion tons in 115 years. The Global Carbon Project stated an annual rise of 2.7%, and 0.6% in 2018 and 2019, respectively. Recent trends showed that the global CO_2_ emissions were over 5% lower in 2020 than in 2019, mainly due to an 8% decline in emissions from coal, 2.3% from natural gas and 4.5% from oil triggered by the COVID-19-forced confinement.^5,6^ This reveals that CO_2_ emissions coupled with energy and industrial production occur from various fuel types (coal, oil, gas, flaring, and cement production).

The advancement of innovative infrastructures for a cleaner energy source (solar energy, hydrogen, wind, or nuclear power) as a long-period platform can reduce CO_2_ emissions. However, so far, renewable energy usage has not reached a level where it can make a considerable contribution to emissions reduction.^7–9^ From past few decades, both industry and scientific community focused on carbon capture and storage (CCS)^10–12^ and carbon capture and utilization (CCU)^13–17^ as part of the mitigation program. To reach their goals, the key part is CO_2_ capture, selectively from the gas mixture. There are various technologies to capture CO_2_, including chemical absorption, membrane separation, adsorption, and cryogenic CO_2_ capture. The traditional method of absorption by liquid amines,^18^ and some self-claimed green solvents including task-specific ionic liquids (ILs), amino acid-functionalized ILs, IL-mixed solvents, and deep eutectic solvents, prove to be highly conventional and are proposed as promising materials. Yet, despite their advantages, they suffer from serious corrosion problems,^19^ considerable energy loss, high sorbent cost, lower gas–liquid interfacial surface area, and ineffective regeneration. Due to these limitations, CO_2_ adsorption by solid porous material presents advantages such as high uptake efficiency, easy recovery, and high adsorption capacity under humid conditions, easy handling, and material stability.

**Fig. 2:**
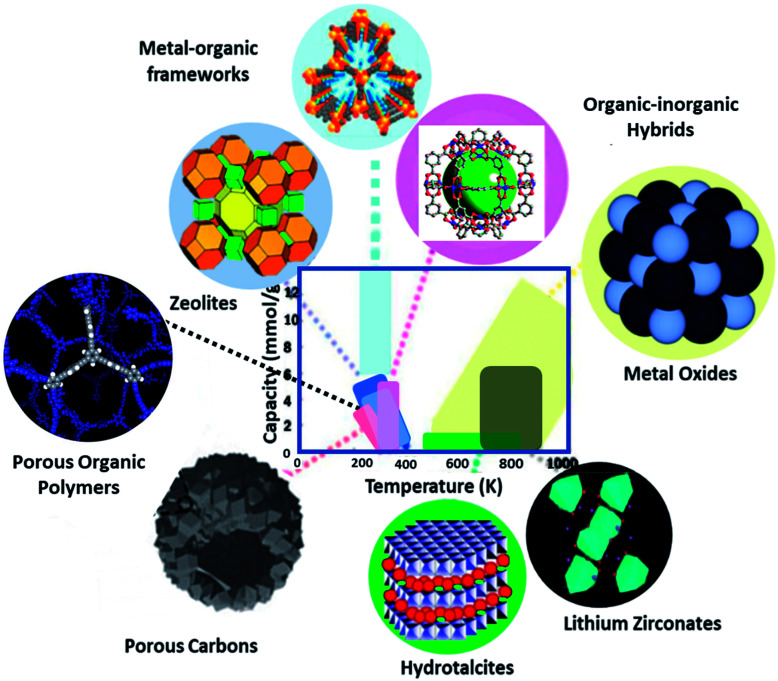
Potential porous solid adsorbents and the relevant relationships between capacity and temperature (data from ref. 20).

Presently, a wide variety of solid adsorbents has been employed for CO_2_ capture,^20^ which comprises metal–organic frameworks (MOFs), zeolites, mesoporous silica, clay, porous carbons, porous organic polymers (POPs), hydrotalcite, organic–inorganic hybrids, and metal oxides. These solid porous materials have different physicochemical interactions with CO_2_ molecules. However, by seeing Fig. 2, one can understand the operating range of these sorbents based on adsorption–desorption temperatures. MOFs, zeolites, silica, clay, POPs, carbons, and hybrids were performed at the low temperature (<473 K) region. While the metal oxides and hydrotalcite react at intermediate (473–673 K) to a high-temperature range (>673 K), lithium zirconate performs in the high-temperature range. Knowledge of the physicochemical features leading to solid adsorption is a criterion for improving gravimetric and volumetric CO_2_ uptake. In the development of new adsorbents, one should contemplate using bigger pores (tens of nanometers) to enable fast transport and gas/surface interactions, and even possibly tunable nanopores to assist both uptake and discharge of targeted gases. The functionalization of these adsorbents also improves the performance of CO_2_ capture. This review focuses on the most relevant and advanced aspects in the field of porous solid adsorbents design, preparation and surface modifications, and subsequent assessment in the CO_2_ adsorption process, accompanied by their possible disadvantages and advantages associated with them.

## 2. Porous materials

### 2.1. Metal–organic frameworks (MOFs)

Metal–organic frameworks (MOFs) likewise called coordination networks or Porous Coordination Polymers (PCPs) (an additional condition that porosity needs to be proven) are a class of crystalline porous adsorbents (or is a coordination network with organic ligands comprising potential voids) that are believed to be of huge potential in CO_2_ capture applications. As per the Cambridge Structural Database (CSD) estimation, more than 90 K structures are existing in its MOF subset.^21^ MOFs are composed of organic bridging ligands and inorganic nodes (metal-based) linked *via* a coordination bond to produce a 3D expanded network with a consistent pore diameter in the range of 0.3 to 2 nm. The nodes commonly comprise single or multi-inorganic nodes/metal ions (for example, Mg^2+^, Cr^3+^, Zn^2+^, Cu^2+^, or Al^3+^, *etc.*) to organic ligands bridging coordinatively *via* a certain functional group (*e.g.*, pyridyl, carboxylate, *etc.*). MOF worldwide market is projected to expand at a CAGR of nearly 34.3% over the next five years and is expected to reach 410 million USD in 2023, from 70 million USD in 2017. Owing to their advantages such as their extraordinary surface areas reaching a further 6000 m^2^ g^−1^, and ultrahigh porosity (up to 90% free volume), their performance is outstanding. One of the desired benefits of these frameworks over other adsorbents is the flexibility to tune the pore size and surface modification by a sensible variety of the organic bridging ligand, activation method, metal ion, and functional group.^22–25^ Yaghi *et al.* first forecasted the probability of MOFs in 1998 as CO_2_ adsorbents and observed one of the MOFs (Zn (BDC)) with a CO_2_ capture of 2 mmol g^−1^ at 195 K and 1 bar pressure.^26^ Subsequently, many scientists have developed and observed many MOFs for CO_2_ adsorption.

Compared to zeolites (discussed below), MOFs processed much CO_2_ uptake at moderate pressures owing to their elevated surface area to weight ratio. While zeolites dominate better adsorption capabilities at low pressures (<10 bar) and high pressures (>10 bar), it is expected that their full capabilities are constrained to one-third of individual metal–organic frameworks. When comparing the active surface area per unit weight: MOFs are in the range of 1500–6000 m^2^ g^−1^, whereas the activated carbons are in 400–2000 m^2^ g^−1^ and zeolites up to 1500 m^2^ g^−1^.^27^ It has been observed that CO_2_ adsorption is a relative function of pressure in the gas phase, whereas low pressure resembles post-combustion techniques. The gravimetric adsorption of CO_2_ is an analytical to the capacity of frameworks to capture CO_2_. Numerous approaches have been implemented for the progress achieved from the performance of MOFs towards enhancing the CO_2_ adsorption and selectivity. These may include open metal sites, pre-synthetic modifications such as phosphonate, amine, and sulfonate functionalization, multi-functional ligands, mixed ligand-functionalization, open nitrogen sites in the framework and post-synthetic techniques like ethylenediamine functionalization.^28,29^ Recent usage of amalgam structures centered on MOFs with other solid adsorbents like activated carbon, graphene, graphene oxide (GO), and carbon nanotubes (CNTs) offer the additional aspects of greater surface area and effortlessly-functionalized sites for modification of definitive composite material properties.^28^ Yang *et al.* stated that Mg-MOF-74 crystals comprising of Mg^2+^ sites with an open framework with a surface area of 1525 m^2^ g^−1^ prepared by sonication showed a greater CO_2_ capture of 350 mg g^−1^ at atmospheric ambiance.^30^ As MOF-74 holds open metal sites (Lewis acidic sites), CO_2_ is performed as Lewis base strongly bound with the open metal sites.

**Fig. 3:**
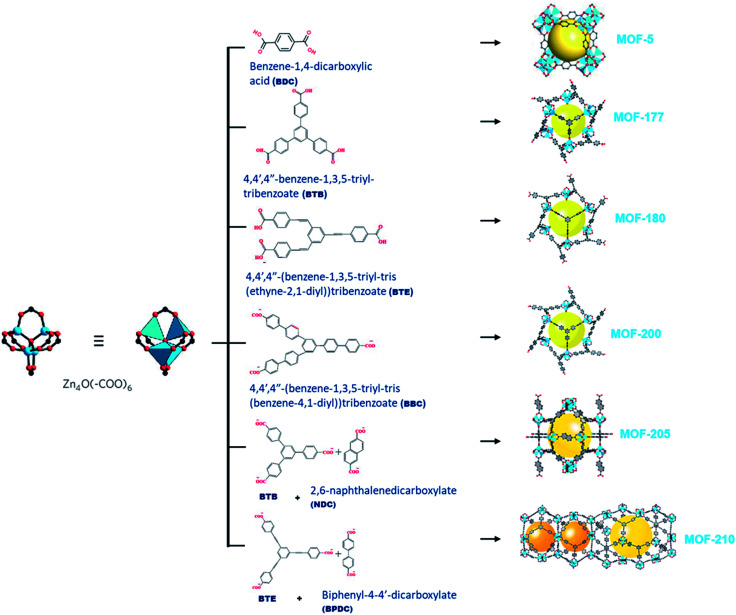
An illustration of a Zn_4_O(–COO)_6_ unit (left) is linked with organic linkers (middle) to shape different types of metal–organic frameworks (right).^34^

MOF-177 possesses a surface area of 4500 m^2^ g^−1^ that displays CO_2_ capture up to 33.5 mmol g^−1^ at 32 bars.^31,32^ The octahedral Zn_4_O(–COO)_6_ building unit, containing single or double organic linkers will form different MOFs exhibiting exceptional porosity as shown in Fig. 3 and the organic link 4,4′,4′′-benzene-1,3,5-triyl-tribenzoate (BTB) in MOF-177 by exchanging bigger 4,4′,4′′-(benzene-1,3,5-triyl-tris(benzene-4,1-iyl))tribenzoate (BBC) link; MOF-200 attained more surface area than MOF-177. This demonstrated the capacity of CO_2_ capture of ∼71 wt% at 50 bar and 298 K.^33^ Furukawa *et al.* prepared ultrahigh porous MOFs with Zn_4_O(–COO)_6_ unit. Amongst them, MOF-210 exhibited an elevated pore volume of 3.6 cm^3^ g^−1^ and the highest reported BET surface area of 6240 m^2^ g^−1^ compared with 4530 m^2^ g^−1^ for MOF-200. This ultrahigh porosity is primarily attained by expanding organic linkers and is close to the theoretical limit for adsorbents.^34^ Both MOF-200 and -210 showed CO_2_ adsorption of 2400 mg g^−1^, which surpasses those of MOF-177 and MIL-101c(Cr) porous adsorbents with 1470 mg g^−1^ and 1760 mg g^−1^, respectively.^35^ This maximum CO_2_ uptake is proportional to the excess pore volume in their structures.

**Fig. 4:**
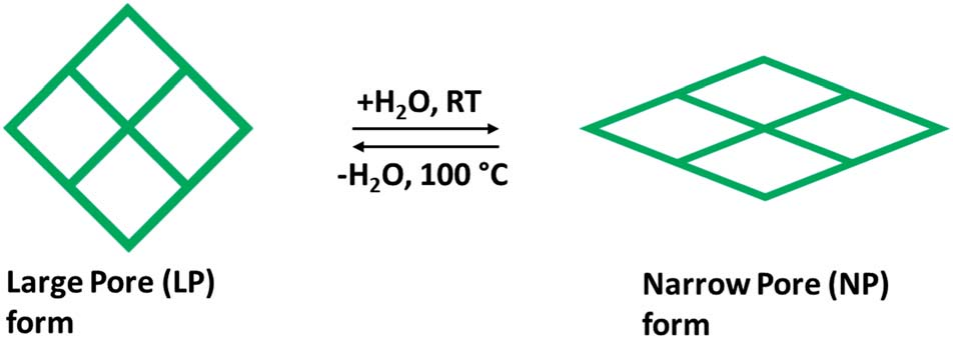
Demonstration of the breathing effect of flexible MOF structure containing LP with almost rectangular pores, and NP with trapezoidal pores.

Rigid MOFs usually have stable and robust porous frameworks with permanent porosity. Compared to such rigid frameworks, flexible frameworks restore their porosity upon adsorption and desorption due to breathing motion, and they are characteristically affecting a volume change of 50–85%. During the ejection of guest solvent molecules, a collapse of the flexible and dynamic frameworks takes place but retain their porous structures by high-pressure adsorption (external stimuli). The design and preparation of a flexible framework reckon the selection of the framework elements as organic linkers and metal nodes. This one has significance in stating that the selection of metal centers has been built partly on the framework structure procedure. Though, organic linkers holding functional groups are getting further interest to perform the main part of MOFs flexibility.^36^ The sequence of metal terephthalates MIL-53 or M(OH)(O_2_C–C_6_H_4_–CO_2_), where M = Cr^3+^, Al^3+^, or Fe^3+^, are one of the best remarkable examples due to their ability to tune shape and pore size to acknowledge linear hydrocarbons, adsorption of polar molecules (CO_2_, H_2_O) and did not change any structural amendments when adsorbing light hydrocarbons (methane) and further small nonpolar molecules. The existence of breathing upon adsorption relays on the pore volumes and the variation in free energy in the rectangular large pore (LP) and narrow pore (NP) with trapezoidal shape in the host structure as shown in Fig. 4, as well as the adsorption affinities of both structural forms (LP and NP). Some of the MOFs, such as MIL53(Cr), MIL-88 that fit into third-generation porous adsorbents, remarkably demonstrate intense modifications of shape when guest molecules are incorporated or separated and a breathing framework with flexible and dynamic properties. These properties significantly promote gaseous molecular adsorption and delivery performance. Besides this, selectivity is a critical aspect related to CO_2_ separation. This can be explained by using flexible porous chromium terephthalate represented as MIL-53(Cr) when the capture of CO_2_/CH_4_ combination is exposed. The effect of breathing is largely contributed by the CO_2_ partial pressure with methane taking a slight effect on the transition level among the open and closed structures.^37^ The existence of water steers a strong rise in CO_2_ capture compared to CH_4_ in the MIL-53(Cr).^38^ Overall, for preparing flexible frameworks, the introduction of particular linkers or functionalities, metal ions/nods are not sufficient, but also the perceptive functionalization and mild interaction must occur among them, in terms of distance, location, and concentration of distinct framework elements. Thus, it is anticipated to observe certain parallelism among the influences of organic linkers, metal nodes, and in what manner the whole framework can react to any differences in pressure, temperature, or guest molecules (external stimuli).^36^

The inclusion of heteroatoms inside the structure or as an element of a covalently bound functionality, particularly individuals in which they acquire superior polarity and, in a few situations, a nucleophilic nature, has proven huge potential for presenting sharp interactions with CO_2_.^39^ Functional groups grafting along with a high CO_2_ affinity on the porous adsorbent's surfaces *via* ligand alteration, or coordination to unsaturated metal centers are utilized as an approach to improve CO_2_ adsorption selectivity and capacity. This method has correlations through many functionalized porous materials like silica grafted with amines; nevertheless, the crystalline form of MOFs delivers molecular stage management in tuning the gas separation properties. Functionalities on the organic bridging ligands, comprising F, Cl, Br, Cl, NO_2_, CN, and NR_2_ groups, can improve the electronic interaction with CO_2_. When comparing with other stated frameworks having derived Lewis acid sites as coordinatively unsaturated metal centers, MOFs with impregnated Lewis base are occasionally seen due to the propensity of Lewis basic sites to coordinate metals during the preparation of MOF. A novel high micropore and 3D nitrogen-rich units containing Lewis acid–base bifunctional Zn(ii)-based MOF-Zn-1 [Zn_2_L_2_MA·2DMF] (MA = melamine, H_2_L = 2,5-thiophenedicarboxylic acid), with a surface area of 1006.3 m^2^ g^−1^ show an enhanced CO_2_ uptake of 4.82 mmol g^−1^ at 273 K, which is higher than that observed for numerous reported metal–organic frameworks. For instance, USTC-253, NTU-105, and [Cu_3_(cpbda)_2_(H_2_O)_3_](DEF)_4_, with BET surface areas of 1800 m^2^ g^−1^, 3543 m^2^ g^−1^, and 1926 m^2^ g^−1^, displayed CO_2_ capture of 3.67 mmol g^−1^, 4.2 mmol g^−1^, and 4.55 mmol g^−1^ at 273 K, respectively.^40^ Ding *et al.*^41^ used imidazolium-type polyionic liquids referred to as polyILs that have been threading into the MIL-101 (Cr_3_X(H_2_O)_2_O(BDC)_3_·*n*H_2_O). The obtained polyILs@MIL-101 composite with a pore volume of 1.26 cm^3^ g^−1^ and BET surface area of 2462 m^2^ g^−1^ displayed good CO_2_ capture capabilities of 2.76 mmol g^−1^ at 298 K and 4.6 mmol g^−1^ at 273 K at 1 bar pressure, mainly credited to the polyILs addition which causes the creation of extra tiny pores (<0.8 nm). The considerably improved capacity of polyILs@MIL-101, related to both polyILs and MIL-101 framework, is credited to the synergistic effect between the Lewis acid sites in MOF, along with Lewis base sites (Br^−^) in polyILs, and better CO_2_ supplementation capacity.

The dialkylamines grafting or incorporation within MOFs have also been represented as a favorable way to enhance CO_2_ adsorption. The improvement of these structures is due to the chemisorption way of CO_2_ adsorption, these usually result in better CO_2_ adsorption and selectivity at low pressures as of flue gas.^42^ Besides, alkylamine functionalities surmount the problem of viable CO_2_ capture in the existence of water. Primarily the grafting of dialkylamines in frameworks was stated in 2008 utilizing MIL-101(Cr) (Cr_3_(F, OH) (H_2_O)_2_O(BDC)_3_·*x*H_2_O; BDC = terephthalate), where amines are attached to Cr.^43^ Later reports displayed the adsorption of CO_2_, in which ethylenediamine (en) was grafted onto Cu^2+^ in the sorbent comprised Cu_4_Cl SBUs (secondary building units) connected by tritopictriazolate with one non-coordinating nitrogen atom.^44^ This displayed that the ethylenediamine-functionalized CuBTTri which has 1.6 wt% CO_2_ adsorption surpasses the original framework containing 0.92 wt% CO_2_ capture at 298 K and ∼0.06 bar.

Consequently, the amine affinity on CO_2_ has led to the incorporation of amine-functionalized interest in numerous MOFs to improve the adsorption uptake and its selectivity. When comparing the improvement of CO_2_ adsorption to methane for amino-MIL-53(Al), which is [Al(OH)(NH_2_bdc)] contrasted with the original MIL53(Al), a flexible MOF.^45^ Additionally, alkylamine-functionalized MOFs were demonstrated to improve the CO_2_ adsorption selectivity, particularly at the lower pressures applied to the separation of flue gas.^46^ A stable amide-functionalized MOF, including the prominence of greater selectivity of CO_2_/N_2_ and high physiochemical stability, in this AFMOF as [Sc_3_(μ^3^-O)(L)_1.5_(H_2_O)_3_Cl]_*n*_ [NJU-Bai49; H_4_L = 5-(3,5-dicarboxybenzamido)isophthalic acid], which showed the uptake of 4.5 wt% CO_2_ at 298 K and 0.15 bar, and greater CO_2_/N_2_ selectivity (166.7) with several AFMOFs owing their performance mainly to the amide functionality and open metal binding sites.^47^

Lately, the addition of alkyldiamines coordinative to unsaturated metal sites padding the pores of the chosen MOFs was established as a straightforward approach to boosting low-pressure CO_2_ capture capacity and selectivity. Significantly the functionalization of Mg_2_(dobpdc) (dobpdc^4−^ = 4,4′-dioxidobiphenyl-3,3′-dicarboxylate), an extended optional MOF of the well-considered MOF Mg_2_(dobdc) (dobdc^4−^ = 2,5-dioxidobenzene-1,4-dicarboxylate), with *N*,*N*′-dimethylethylenediamine (mmen) produced a material having excellent CO_2_ capture in flue gas environments and produced rare and baffling step-shaped adsorption isotherms. McDonald *et al.* and his group interpreted a unique mechanism offering rise in step-shaped isotherms, alongside validating the substituting Mg^2+^ with additional bivalent metal ions. This phenomenon allows the displacement of the carbon dioxide capture step to be engineered as per the agreement of the strength of the amine-metal bond. Like this has been demonstrated, the family of diamine-appended MOFs such as mmen-M_2_(dobpdc) compounds, where M = Fe, Mg, Co, Ni, Mn, Zn, *etc.*, are better materials than many liquids or solid sorbents for effective CO_2_ adsorption.^48^

Kim *et al.*^49^ developed a group of tetraamine-appended magnesium MOF that demonstrated supportive CO_2_ capture and highly improved stability associated with earlier-noted diamine-appended frameworks due to multiple, ordered metal–amine interactions. In this case, a hexagonal channel of Mg_2_(dobpdc), diamine-functionalized material features coordination of one diamine to each Mg^2+^ site,^50^ whereas tetraamines can coordinate to two Mg^2+^ sites. The type of amine functionality on these frameworks enhances two-step CO_2_ capture and significant adsorption enthalpies appropriate to simulated streams of natural flue gas for CO_2_ adsorption. The high-performance sorbent, Mg_2_(dobpdc) (3-4-3), attained a greater carbon dioxide capture of 2.0 ± 0.2 mmol g^−1^ in the ambiance of water, whilst reaching the objective of DOE to adsorb 90% carbon dioxide from natural gas flue emissions. Very importantly, the improved stability of these tetraamine-appended MOFs would adsorb CO_2_ from wet air and can be stimulated with steam, this approach is highly cost-efficient than pressure or temperature swing routes.

**Fig. 5:**
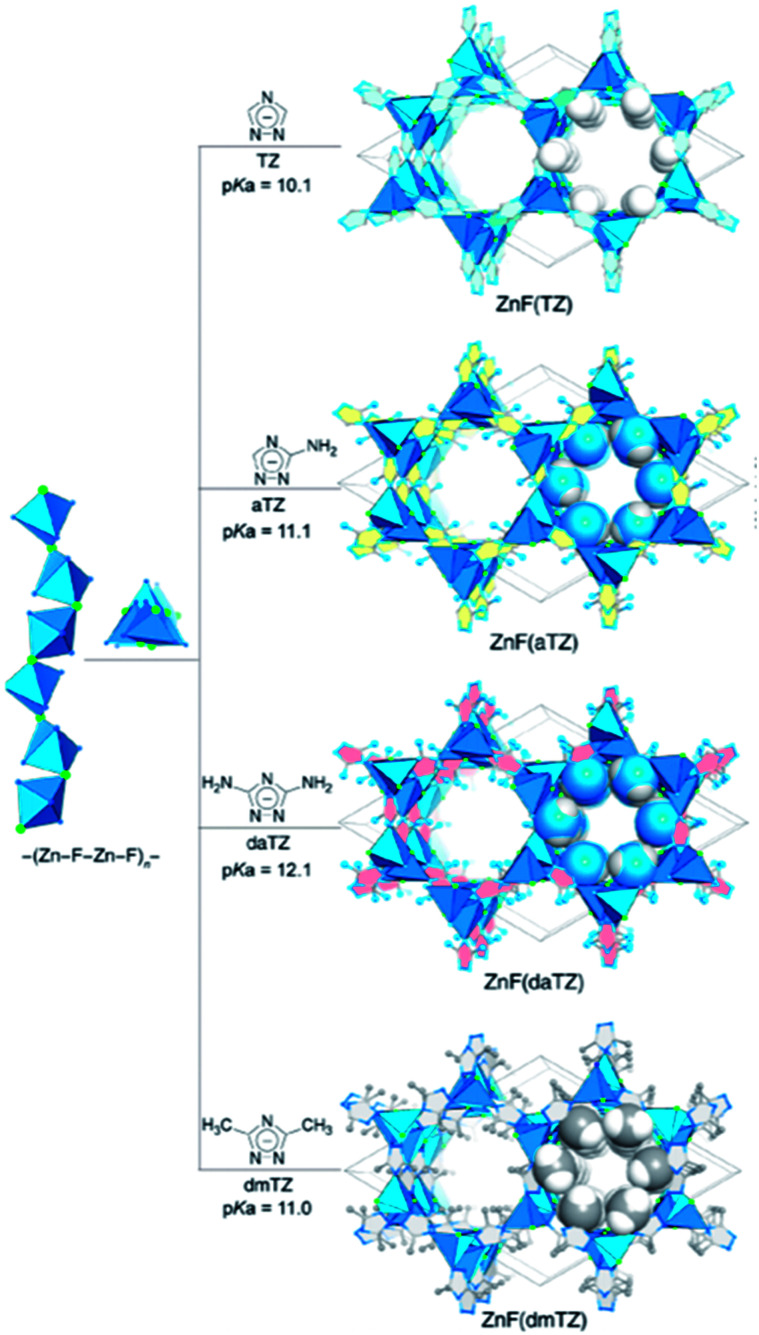
Graphical illustration for the framework construction of a robust isoreticular metal triazolate (Reproduced with permission from ref. 51 Copyright© 2020, American Chemical Society).

Recently, interest in constructing a novel group of water and base stable MOFs has grown due to their advantages of geometric rigidity, strong affinity towards CO_2_ binding, and electrostatics. Azolate linkers, such as 1,2,4-triazolate (TZ), 1,2,3-triazolate (Tz), and imidazolate (IM) have been used to prepare robust MOFs. Shi *et al.*^51^ used robust isoreticular metal triazolate frameworks with excellent CO_2_ adsorption under humid conditions. ZnF(TZ) and its isostructures are shown in Fig. 5 constructed by connecting TZ struts to –(Zn–F–Zn–F)_*n*_– SBUs rods, resulting in a honeycomb-like channeled framework [ZnF(TZ), ZnF(aTZ), Zn(daTZ), and ZnF(dmTZ)] with high thermal and chemical stabilities, which also can be modified by the functionality on the TZ struts. Additionally, the thermodynamic (CO_2_/N_2_) and kinetic (CO_2_/H_2_O) adsorption selectivities can be modified by the number of functional groups on the struts. Among them, ZnF(daTZ) showed the maximum CO_2_ adsorption of 0.96 mmol g^−1^ at 0.15 bar with high CO_2_/N_2_ (85 : 15) selectivity (120) and H_2_O/CO_2_ selectivity (3000) in the humid region.

**Fig. 6:**
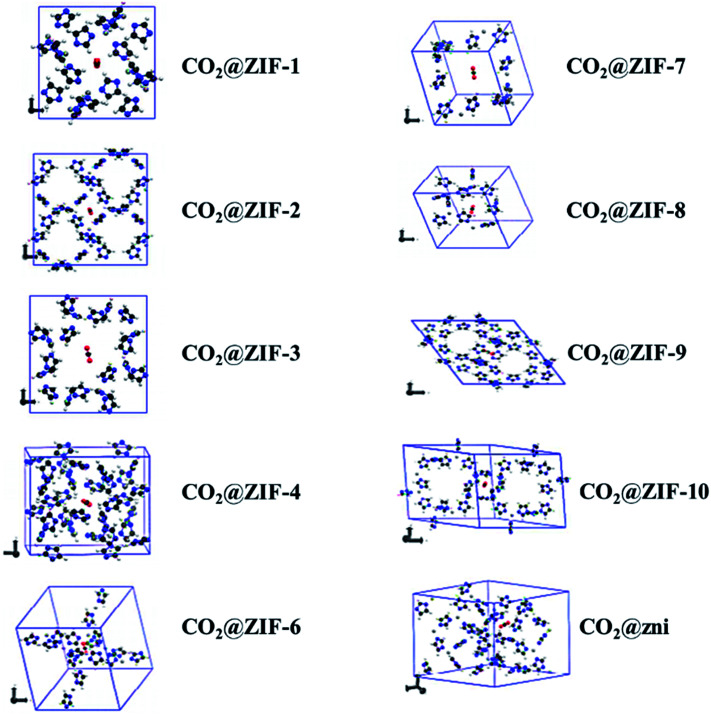
Optimized geometries of the different CO_2_@ZIFs complexes where the CO_2_ molecule is trapped in the cavity center of each ZIF structure (reproduced with permission from ref. 53. Copyright© 2017, American Chemical Society).

**Fig. 7:**
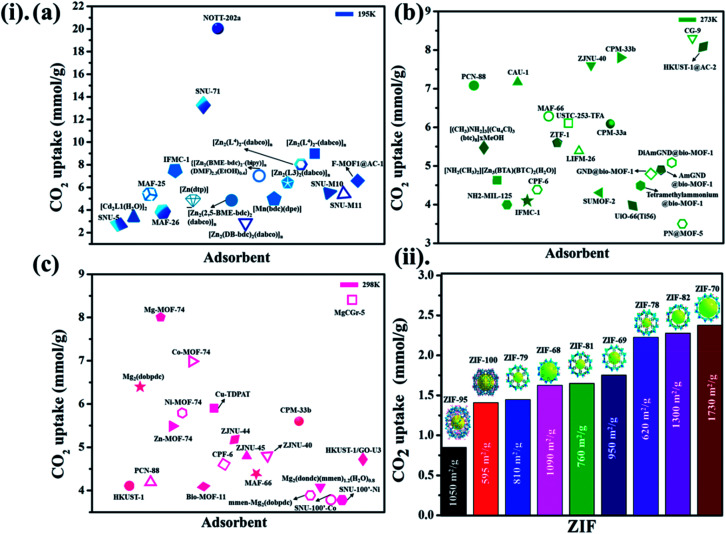
(i) The literature on different MOF-based adsorbents for CO_2_ uptake at different temperatures (a) 195 K, (b) 273 K, (c) 298 K for 1 bar pressure (for some adsorbents CO_2_ uptake units are converted from the originally reported ones). (ii) Demonstrates a series of ZIFs along with their surface area towards CO_2_ uptake at 273 K.

Not long ago, researchers demonstrated that Si–O–Si favored angle in zeolites (145°) as an equivalent to that of the M–Im–M fragment bridging angle (where M is Zn or Co and Im is imidazolate), and consequently, it is probable to prepare new zeolitic imidazolate frameworks (ZIFs). ZIFs are a subclass of MOFs, which are topologically isomorphic with zeolites that comprise of a tetrahedral cation coordinated by an organic imidazolate (IM) bidentate ligand or substituted derivative thereof. ZIFs produce stable, highly crystalline, and 3D crystalline microporous solids with strong adsorption sites. Owing to their large interior pores (3–12 Å) and porous nature, ZIFs have exceptionally minimal density and high surface areas in the range of 1000–2000 m^2^ g^−1^. ZIFs acting as selective CO_2_ adsorbents eventually rely on their specific interactions with CO_2_ molecules and show greater selectivity than MOFs for CO_2_ from other relevant flue gases (such as CO).^52^ Theoretical results showing CO_2_ adsorption by distinct sets of ZIF adsorbents (ZIF-1 to -4, -6 to -10, and -zni) are illustrated in Fig. 6 and the calculated outcomes of the dipole moment variation, the interaction energy, and the charge density variation for the various CO_2_@ZIF forms are also presented. It demonstrated a solid correlation between the ZIFs cavities volume and CO_2_ adsorption energy: the CO_2_ uptake relies on the size and shape of its pore where gas molecules have been entrapped. The physisorption mechanism that controls the carbon dioxide capture expects combined hydrogen-like bonding and π-stacking interactions. They also, concluded that the adsorption does not alter the geometry of CO_2_. However, it stimulates a major structural difference in some ZIFs.^53^ Phan *et al.*^54^ used a series of ZIFs (ZIF-68, -69, -70, -78, -79, -81, -82, -95, and -100) to study their surface area, CO_2_ uptake at 273 K in the low-pressure regions as shown in Fig. 7(ii), though their affinity to it is not always strong. In that, 68, 69, and 70 indicated high CO_2_ affinity.

While polar functional groups benefit from stronger CO_2_ interactions with enhanced selectivity and adsorption capacity, establishing hydrophobicity is a substitute for CO_2_ adsorption by an easy elimination of water from the pores.^55^ Usually, the selective adsorption of CO_2_ for MOFs that merely depend on hydrophobicity over hydrophilicity endure weak adsorption (due to an absence of solid binding sites for CO_2_) in contrast to MOFs with other structural aspects. These hydrophobic MOFs are a substantial move in a suitable route for employing them in workable circumstances, there persists an evident lack of linking all encouraging fundamental aspects as a single framework. The coordination of heteroatoms or unsaturated metal sites in MOFs makes them water shielded as per hydrophobicity. It is worth noting that by observing many experimental and simulation studies, the adsorption enthalpy plays a major role together with the free volume and surface area of a material. At higher pressures, the surface area and free volume in adsorbent perform major responsibility in CO_2_ capture, while at low pressures, enthalpy of adsorption correlates more. Undeniably, still, there is a persistent demand for a deeper experimental knowledge of MOF and carbon dioxide connections to define the structural characteristics accountable for efficient CO_2_ adsorption performance.^56–58^Fig. 7(i)(a–c) presented the different MOF-based adsorbents for CO_2_ uptake at different temperatures (195 K, 273 K, 298 K) for 1 bar pressure.^59–86^

### 2.2. Zeolites

Zeolites are crystalline solid structures rendered of a TO_4_ tetrahedra periodic array, in which T signifies Al or Si. Every oxygen is linked to four T-atoms towards building perfectly characterized channels and pores with sizes in the range of 5 to 12 Å. These are also considered molecular sieves because of their microporosity. A variety of zeolite structures have been reported previously, guiding broad flexibility in their pore dimensions, channel system dimensionality, or composition, which makes zeolites one of the attractive materials of CO_2_ adsorption. Though natural zeolites (clinoptilolite) are available, these materials have also been synthetically produced (Type-A (LTA), Type-X, Type-Y, USY, and ZSM-5, *etc.*), due to huge flexibility to control their porosity and crystallinity. The existence of more aluminum content in the silicate-framework enhances zeolite basicity. The result is mainly caused by a lower electronegativity of aluminium related to silicon. Higher the zeolite basicity, the better is the CO_2_ capture. The overall zeolite market is estimated to grow US $11.2 billion in 2020. Zeolite's CO_2_ adsorption capacity is significantly better at room temperature than at elevated temperatures. The CO_2_ capture considerably increases with a slight reduction in adsorption temperature under a fixed pressure. This was further discussed by observing 13X, 5A, 4A, WE-G 592, and APG-II demonstrating the CO_2_ capture at 393 K. Nevertheless, CO_2_ uptake is less at 393 K compared to room temperature.^87^ Some factors need to be considered while evaluating the effectiveness of the zeolite towards CO_2_ adsorption. The primary one is basicity, which is produced by executing an alkali metal cation exchange. The following is the ratio of Si/Al that affects the cation exchange capacity. To obtain a better uptake, there needs to be a lower Si/Al ratio to improve the cation exchange capacity.

**Fig. 8:**
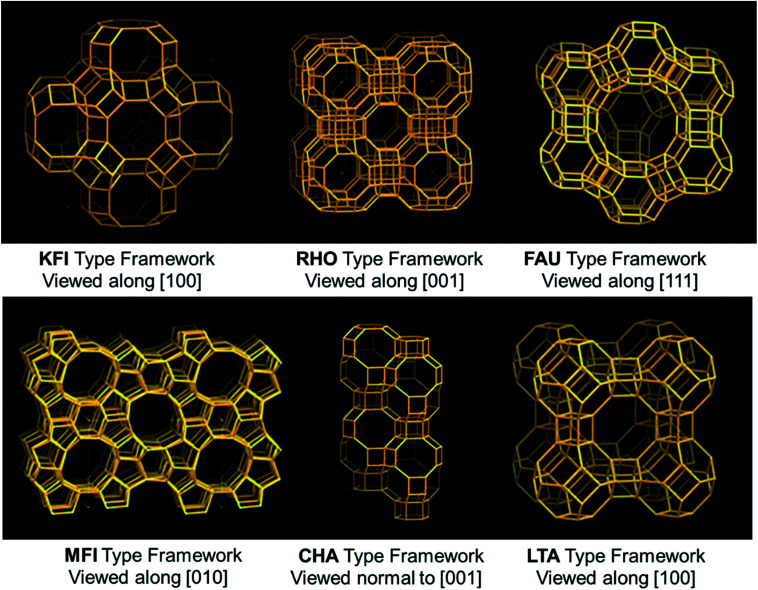
Some of the frameworks of zeolite structures (KFI, RHO, FAU, MFI, CHA, and LTA-type structures).^21^

The micro/mesoporous zeolites are counted as capable CO_2_ capture materials since the modification of porous structures to surmount the limitations of molecule diffusion.^88,89^ Liu *et al.*^90^ used highly porous ZSM-5 (MFI-type) prepared *via* a growth-inhibition strategy by organosilanes. The CO_2_ capture of HP-ZSM-5 reached 2.6 mmol g^−1^ at 273 K under atmospheric pressure, which is considerably more than that of ZSM-5. This enhanced capacity of CO_2_ capture is primarily by the intricate surface morphology and micro-/mesoporous composite structure that results in a porous network with a low-resistant route for CO_2_. Besides, ZSM-5 has a high affinity towards CO_2_ molecules due to interactions arising from the electric field of ZSM-5 and a quadrupole moment of CO_2_. Kongnoo *et al.*^91^ used zeolite 13X, which is prepared by acid activation (HCl) of the palm fly ash to improve its CO_2_ capture by increasing the total pore volume and its mesopores. As such, the activated zeolite 13X showed 22% advanced (6.42 mol CO_2_ per kg) adsorption capacity than those of the unactivated ones and is 11% greater than that of the commercially available zeolite 13X at 305 K and 403 kPa. Some of the framework types of zeolite structures used in this review are shown in Fig. 8.

For instance, X- and Y-type zeolites have a broad scope of commercial applications owing to their huge total pore volumes and stable crystal structures. These adsorbents show a similar cage framework; however, X-type has an additional aluminium content (more cations) than Y-type zeolites. The transferable cation is an acid site, and the framework oxygen closest to the cation delivers a basic site. This increased basicity is mainly due to the high aluminum content in the framework.^92^ By simply altering the aluminum content in the framework or exchanging the cations, one can tune the strength of these zeolite acid–base pairs.^93^ For the first time, Krista *et al.* studied CO_2_ capacities and adsorption equilibrium isotherms in Y and X-type zeolites in the sodium form (NAX, and NAY) tuned by an exchange of alkali metal cation (Li, Na, K, Rb, Cs) at 1 bar and 298 K. For X-type, CO_2_ uptake improved in the order of decreasing ionic radii as Cs < Rb < K < Na < Li, and for Y-type as Cs < Rb ≈ K < Li ≈ Na. In these two types, the bigger cation types (Cs, Rb, K) demonstrated strongly nonlinear concave descending isotherms, representing strong interactions between zeolite and CO_2_. This behavior is steady with improved framework basicity related to the smaller cation forms as CO_2_ is a weakly acidic gas.^94^ Similarly, Pham *et al.* described a complete structural and adsorption study of cation sites Li^+^, Na^+^, K^+^, and Mg^2+^ exchanged ZK-5 zeolites and revealed CO_2_ capture sites within the ZK-5 framework (KFI-type). Li-ZK-5 exhibited high CO_2_ capture at 1 bar pressure than other forms.^95^ Furthermore, Sun *et al.*^96^ studied the significance of transition metal cation-exchanged SSZ-13 zeolites (CHA-type) for CO_2_ adsorption. This process was assessed by unary static isothermal adsorption and binary dynamic column breakthrough tests including expected performance in PSA/VSA process. When comparing the studied transition metals (Ni, Zn, Cu, Co, Fe, Ce, La, and Ag) replacement in SSZ-13, Co^2+^/SSZ-13, and Ni^2+^/SSZ-13 revealed maximum carbon dioxide capture of 4.49 mmol g^−1^ and 4.45 mmol g^−1^, accordingly, and excellent selectivity of CO_2_/N_2_ as 52.55 and 42.61, respectively at 1 atm pressure and 273 K, compared to the remains. This is due to the Π-back donation entirely molded among transition metal cation and CO_2_ molecule.

In contrast, with several zeolites probably vulnerable to CO_2_ capture, RHO-type zeolites are of huge significance owing to their specific 3D structures containing cages and small pore openings. Confalonieri *et al.*^97^ stated that the uptake of CO_2_ is mostly connected with the sodium content in the nanosized RHO crystals. The adsorption tests demonstrated that 1 bar CO_2_ is enough to saturate RHO samples and observed that there was no change in 5 bar pressure at room temperature.

The CO_2_ capture by zeolites has been enhanced by the incorporation of amine moieties into its crystal structure. A few low-cost zeolites, such as zeolite SAPO-34 (CHA-type), Y (FAU-type), and ZSM-5 (MFI-type), have been prepared from kaolin clay for CO_2_ adsorption from the air. Further functionalization (amine impregnation) of these molecular sieves by TEPA enhances the capacity of CO_2_ adsorption. The attained kaolin-based zeolites showed parallel features compared to zeolites prepared with other sources. Besides, these zeolites demonstrated a bi-modal pore network containing both mesopores and micropores. The influence of amine loading on the CO_2_ adsorption revealed that zeolite Y with 10 wt% TEPA showed an enhanced CO_2_ uptake of 1.09 mmol g^−1^ than others, anticipated to its larger mesopore volume.^98^ Fengsheng *et al.*^99^ used TEPA modification of (amine-functionalized) Y-type zeolite with a Si/Al molar ratio of 60 [Y60(TEPA)zeolite] to achieve a significant increase in the capacity of CO_2_ uptake. It was stated that the uptake capacity improved with a temperature between 303–333 K, but diminished within 333–343 K. The phenomenon of Y60 CO_2_ capture is completely a physical interaction, however, after TEPA modification, chemical interaction becomes prominent. Further, the existence of water vapor (7%) enhanced it to 4.27 mmol g^−1^. As described above, Y-type zeolites hold a well-defined pore structure and their pore sizes are of the same magnitude as CO_2_ molecules, thus projected to give a great affinity for CO_2_ adsorption. Murge *et al.*^100^ used zeolite-Y (designated as Z-Y-3, silica to alumina ratio of 2.25) with TEPA modification displayed higher CO_2_ uptake, and the acquired results were about 114 mg g^−1^ and 190 mg g^−1^ for 1 bar and 5 bar pressure, accordingly at 303 K.

Wang *et al.*^101^ used PEI impregnated mesoporous ZSM-5 zeolite prepared from rice husk ash. The measurement of the CO_2_ capture at 393 K revealed that ZSM-5-PEI-30 had a capacity of 1.96 mmol g^−1^, which was nearly 5 times more than the pristine. Pham *et al.*^102^ used ethylenediamine (EDA) functionalized nano zeolite (NZ) to enhance CO_2_ adsorption properties. The CO_2_ uptake of NZ-EDA rose between 293 K to 343 K but then declined further between 343 K to 373 K. The CO_2_ uptake of NZ-EDA was 7.48 mmol g^−1^ at 343 K, which is 2.6 times higher than the NZ sample. As said, after the amine modification, physical interaction turns into a chemical interaction among NZ-EDA and CO_2_ molecules become an important adsorption phenomenon. Chen *et al.*^103^ reported mesoporous zeolite 13X (meso-13X) modified with PEI impregnation and formed a meso-13X-PEI hybrid, which demonstrated a substantial possibility for CO_2_ adsorption capacity. Due to its high pore volume, which permitted for the adjustment of higher amine species than in the case of zeolite 13X. Meso-13X-PEI offered enhanced CO_2_ adsorption of 80 mg g^−1^ than PEI-modified zeolite13X with 48 mg g^−1^ at 373 K. Additionally, Bansiwal *et al.*^104^ analyzed CO_2_ capture utilizing different amine functionalization agents such as DEA, AMP, IPA, MEA, and EDAN on zeolite 13X at various temperatures as 303, 328, and 348 K. The obtained findings supported the above discussion that after amine supplement, zeolite adsorption capacity enhanced-drastically.

**Fig. 9:**
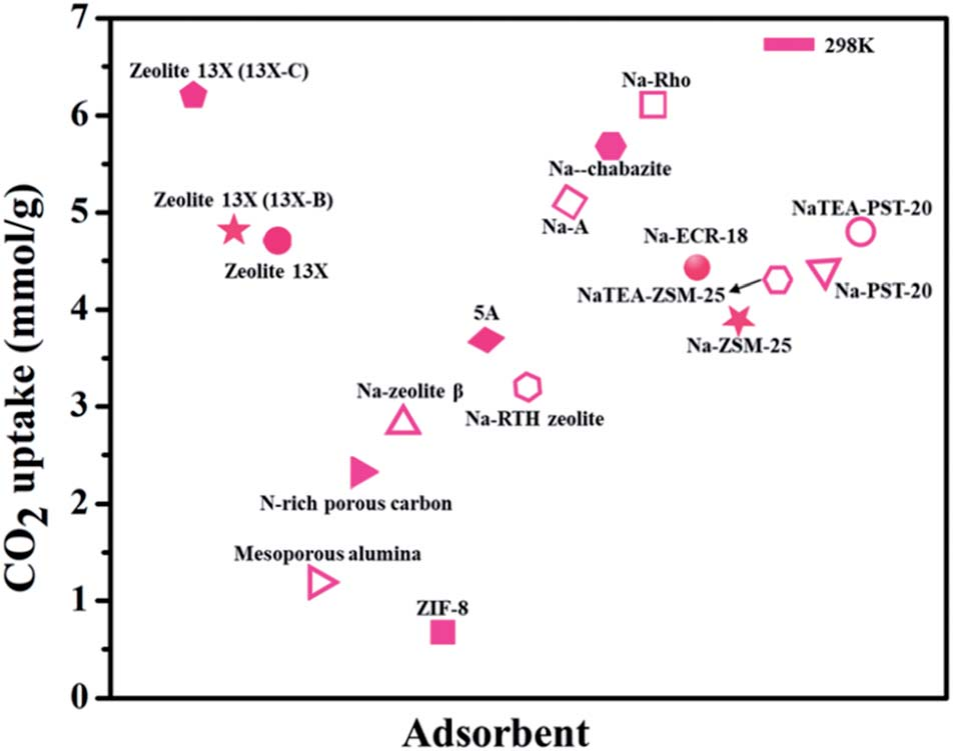
CO_2_ adsorption capacities of zeolite-based adsorbents (for some adsorbents CO_2_ uptake units are converted from the originally reported ones).

It is said that the hydrophilic character of many zeolite structures is contemplated as a main obstacle of zeolites, particularly for post-combustion CO_2_ techniques. The presence of water during CO_2_ uptake on the accessible sorption sites may impact the zeolite framework and its structure. As described above, the existence of uncovered cation sites enhances the capacity of CO_2_ adsorption. The correlation between the water content in zeolite and the cation population density was explored. A direct correlation was observed to explain the reduction of the cation population by raising the quantity of water. This examination underlines the negative impact of the existence of water vapors in zeolites on the capacity of CO_2_ capture.^113^Fig. 9 shows various zeolite-based adsorbents for CO_2_ uptake.^105–112^

### 2.3. Mesoporous silica

Mesoporous silica has attracted great attention after its discovery in the late 1970s. This is mainly owing to their exceptional features, such as ordered pore structures, high BET and preparation in a broad selection of morphologies (powders, discs, rods, and spheres, *etc.*). When compared to conventional porous silica, mesoporous silica shows extremely well-ordered pores. It may be due to the nano templating method employed during the preparation of these adsorbents. Since the last few decades, an overabundance of mesoporous silica adsorbents such as SBA-16, SBA-15 (SBA-Santa Barbara Amorphous), MCM-41, and MCM-48 (MCM-Mobil Composition of Matter), with a variety of pore geometries like cubic, and hexagonal, and morphologies like rods, spheres, and discs have been developed. They are useful in many applications requiring high BET and large pore sizes.^114–116^ Their surfaces containing silanol groups are the key factors. Their functionalization by different organic molecules allows improving their performance in CO_2_ adsorption. The interactions between CO_2_ and the surface of the materials vary, corresponding to the nature of the functionalized molecules or immobilized metals.^117^ The ample hydroxyl groups on the surface of silica extend a prospect of amine functionalization, in other words, enhancing the affinity of CO_2_ and silica interactions with it. Silica-amine compounds have been synthesized either by one-pot synthesis (direct synthesis) or post-synthesis methods. The latter has been utilized highly, where silica is made primarily, and amine species are consequently incorporated into the silica support *via* physical impregnation or chemical grafting.

**Fig. 10:**
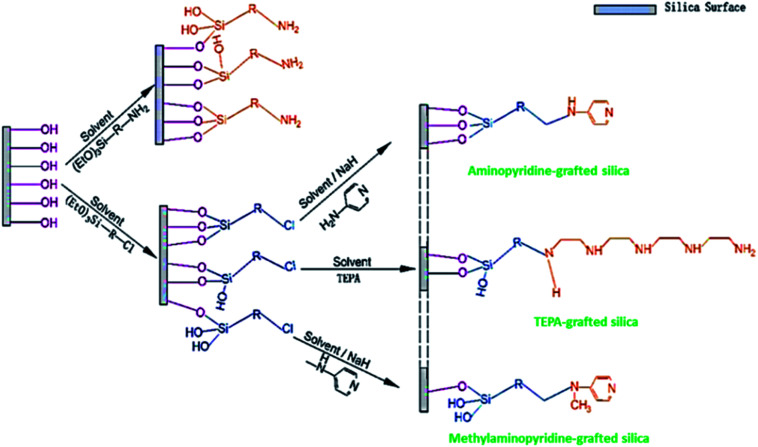
Illustration of amine grafting on mesoporous silica, in which R-denotes an aliphatic carbon chain with or without further secondary amine (reproduced by permission from ref. 118 Copyright 2020, Elsevier).

Based on the amine-loading methods, and the type of bonding between amines and supports will change. In the wet impregnation method, the amine spreads into the support pore networks, extends away on the inside pore surface, saturates the pores progressively, and ultimately spread over the external surface with rising amine content. The formation of the bond between amine and support involves hydrogen bonding. In the grafting method, *via* a chemical bond utilizing silane-coupling agents, amine groups are attached to the silica surface. The linkages formed between various silane-amino species and the support are displayed in Fig. 10. These bonds strengthen the stability of the sorbent whilst constraining the utmost amine content on the support. In sequence, it directs to a shorter capacity of carbon dioxide uptake of amine-grafted one, related to the impregnated adsorbent. Though, insufficient but comparatively constant amine grafting offers a sterically advanced structure for more amine impregnation to deliver high effective sorbents.^118^

**Fig. 11:**
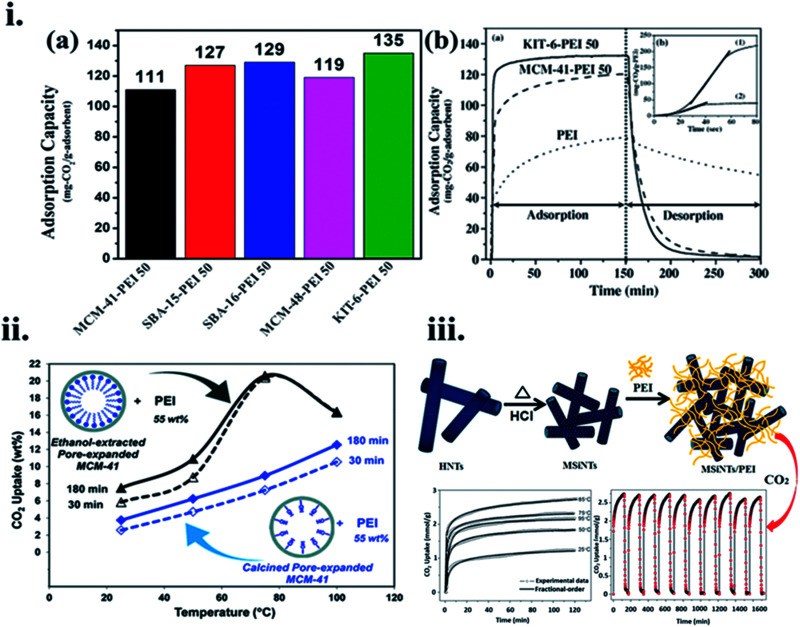
(i) (a) CO_2_ uptake of different mesoporous silica adsorbents after the addition of 50 wt% PEI content and (b). The difference of CO_2_ adsorption–desorption performance between MCM-41-PEI 50, KIT-6-PEI 50, and PEI, inset: assessment of CO_2_ adsorption kinetics of KIT-6-PEI 50 (1) and pure PEI (2). (i) (b) Reproduced by permission from ref. 119 Copyright 2007, Elsevier, (ii) CO_2_ uptake *versus* temperature for PME-PEI (55) after 30 min, and 180 min, PMC-PEI (55) after 30 min, and 180 min of exposure to pure CO_2_ (reproduced by permission from ref. 120 Copyright 2011, ACS publications), (iii) MSiNTs/PEI (MP) nanocomposite preparation and its CO_2_ uptake performance (reproduced by permission from ref. 121 Copyright 2016, ACS publications).

For example, Son *et al.*^119^ used a sequence of mesoporous silica adsorbents, SBA-16, SBA-15, MCM-41, MCM-48, and KIT-6, and functionalized them with 50 wt% PEI loading in methanol to assess the CO_2_ adsorption performance. PEI loaded KIT-6 (6 nm size pores) showed 135 mg g^−1^ CO_2_ adsorption in a stream at 358 K, against a similar amount of PEI loaded MCM-41 (2.8 nm pore size) with 111 mg g^−1^, and their adsorption–desorption kinetics are shown in Fig. 11(i)(a and b) respectively. This can be explained from the fact that silica with a very high total pore volume is more advantageous for amine loading. Similarly, Heydari *et al.*^120^ used PEI-loaded MCM-41 in which the surface is coated with a long-alkyl chain layer initiated to be an additional effective CO_2_ capture material, as shown in Fig. 11(ii). Likewise, when PME had a low BET surface area and total pore volume as 570 m^2^ g^−1^ and 1.59 cm^3^ g^−1^, respectively, compared to PMC with 1254 m^2^ g^−1^, and 2.44 cm^3^ g^−1^, respectively; PME-PEI(55) displayed greater CO_2_ adsorption capacity than that of PMC-PEI(55) at the noted temperatures. PME-based adsorbent showed CO_2_ uptake of 2.3 folds more than its PMC equivalent at 358 K. The distinct performances of these materials were correlated with their pore wall surface. As well as the presence of hydroxyl groups on the PMC surface, instead of long hydrophobic hydrocarbon chains on the surface of PME. Niu *et al.*^121^ used pre-treated pristine halloysite nanotubes (HNTs) to create mesoporous silica nanotubes (MSiNTs) which could be additionally functionalized with PEI to prepare MSiNTs/PEI (MP) nanocomposite with a surface area of at least six times higher than that of HNT, and its CO_2_ capture raised to 2.75 mmol g^−1^ at 365 K as shown in Fig. 11(iii).

Recent literature has demonstrated that some of the amine-functionalized SBA-15 outperforms standard adsorbents such as some of the widely used MOFs and zeolites for CO_2_ sequestration. Kumar *et al.*^122^ reported an amine-modified mesoporous silicate as TEPA-SBA-15, which is a chemisorbent (due to grafting) that relates to a typical group of materials identified as amine-functionalized silica. The TEPA-SBA-15 chemisorbent presents a very high direct air capture (DAC) performance while related to each physisorbents, capturing a great amount of CO_2_ after 12 h of exposure compared to other sorbents such as HKUST-1, Mg-MOF-74, SIFSIX-3-Ni, and zeolite 13X. Zhang *et al.*^123^ used an N-rich SBA-15(p)-AP-70T that showed improved CO_2_ capture of 15.81 mmol g^−1^ and 5.687 mmol g^−1^. Furthermore, uptake stays at 5.2 mmol g^−1^ even after 15 adsorption/desorption cycles, with an associated decline of 6.1%. This is caused by grafted APTES and polyethylene oxide–polypropylene oxide. Polyethylene oxide in the provisional pores might deliver hydrogen-bonding functional groups and spatial partition structure for the distribution of the same quantity of TEPA in the support pore, whereas it decreases the loss of TEPA during the cycle process.

When dealing with porous silica adsorbents, the major obstacle is the degradation of their structure in the ambiance of steam, leading to the emancipation of the grafted or impregnated amines, thus reducing its CO_2_ adsorption capacity along with triggering corrosion complications. Silica frameworks with TEPA and PEI impregnated composites demonstrated enhanced CO_2_ adsorption capabilities at 348 K, owing to the enhanced amine species mobility and CO_2_ diffusion enabled in the pore channels at comparatively high temperatures.^124^ Besides, CO_2_ adsorption by amines is exothermic; accordingly, in the desorption cycle, high temperature is advantageous. Hence, the optimal temperature for the CO_2_ adsorption by TEPA and PEI impregnated compounds were decided by comptonization among the effect of kinetic factors and thermodynamic parameters. The post-combustion temperature of CO_2_ gas is generally between 50–75 °C (ref. 125) and it is near the optimum temperature range of CO_2_ adsorption by PEI or TEPA-impregnated silica composites. Sayari *et al.*^126^ stated the grafting of 3-[2-(2-aminoethylamino) ethyl amino] propyl tri methoxy silane on the pore-expanded MCM-41 surface with an elevated amine content of 6.11 mmol N g^−1^ revealed exceptionally great uptake and adsorption rates as it was subjected to a 5% CO_2_/N_2_ gas mixture. The respective uptakes of 2.65 mmol g^−1^ and 2.94 mmol g^−1^ CO_2_ were attained in a dry atmosphere and humid (298 K, 0.05 atm) ambiance conditions.

### 2.4. Clay-based adsorbents

**Fig. 12:**
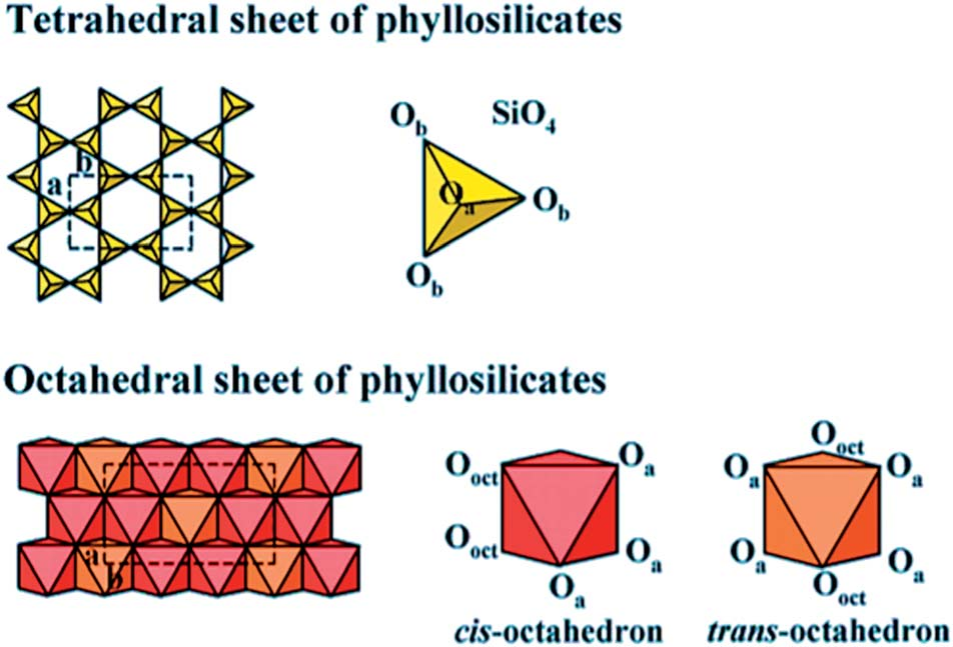
Tetrahedral and octahedral sheets of the phyllosilicates (reproduced by permission from ref. 127 Copyright 2018, Wiley Online Library).

Clay is a usual phrase that specifies a particular clay mineral or a mixture of single or more clay minerals, including small quantities of organic matter and metal oxides. Clays are hydrated aluminum phyllosilicates naturally developed *via* hydrothermal alteration of rocks. Commonly, the structure is composed of tetrahedral and octahedral sheets arranged into layers,^127^ as shown in Fig. 12. The structure additionally contains a different quantity of the large types of cations.^128^ A continuous tetrahedral sheet (T) shaped by [MO_4_]^4−^ types in which M represents Fe^3+^, Al^3+^ or Si^4+^, is located at the tetrahedron center and at the edges four oxygen atoms are situated by connecting to its neighboring tetrahedral by sharing three corners, procuring a 2D form with hexagonal structure along with the *a*, *b* plane. In the octahedral sheet (O), the octahedra are linked by sharing edges, obtaining sheets with hexagonal symmetry with principal cations Fe^3+^, Al^3+^, Fe^2+,^ and Mg^2+^. Owing to cost-effective, plentiful, and moderated porosity, clay-based adsorbents were intended to support commercial CO_2_ uptake. Many studies have explored the clay-based materials as one of the effective adsorbents for CO_2_ capture, including kaolinite, halloysite, bentonite, smectite, montmorillonite, or sepiolite as starting materials.^129–132^ Clay minerals are not conducive to chemical modifications, textural properties can be enhanced, and microporosity can be improved. Additionally, the adsorption capacity of CO_2_ can be increased in many situations. Consequently, the acid treatment of clay boosts microporosity in a limited dissolution of its sheets, supporting these structures to perform as a molecular sieve by adsorbing molecules of CO_2_. The amine impregnation in natural clays improves the capacity of carbon dioxide uptake since there is a rise in the intensity of chemical interactions between the developed compound and CO_2_. This method primarily holds the position on the surface because the amalgamation of the amine in clay blocks the cavities of clay minerals.^133^

**Fig. 13:**
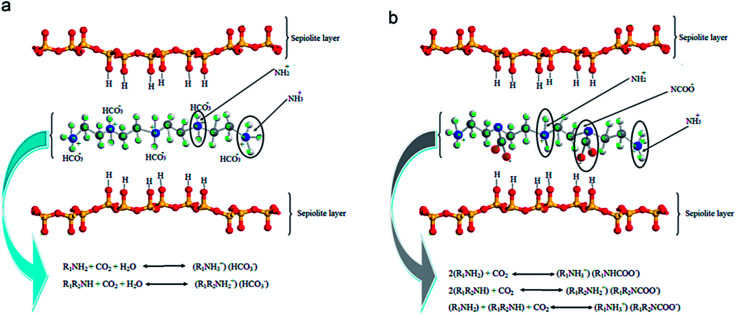
CO_2_ capture mechanisms of (a) humid, and (b) dry conditions (reproduced by permission from ref. 136 Copyright 2014, Elsevier).

Cecilia *et al.*^134^ reported the evaluation of pure CO_2_ capture in a volumetric setup by using two clay minerals such as sepiolite (meerschaum) and palygorskite (attapulgite), which are members of a palygorskite-sepiolite group of fibrous clay minerals. The idealized sepiolite structural formula is Si_12_O_30_Mg_8_(OH)_4_(H_2_O)_8_ is a hydrous magnesium silicate that has eight possible octahedral positions per half unit cell, in which the trioctahedral positions are engaged by Mg^2+^ and minor amounts of Fe^3+^ and Al^3+^. Palygorskite is a dioctahedral mineral with structural formula Si_8_O_20_(Al_2_Mg_2_)(OH)_2_(OH)_4_(H_2_O)_4_, in which the octahedral locations are engaged by Mg^2+^ and Al^3+^ creating voids in the octahedral sheets. In these two, raw sepiolite attained a CO_2_ capture of 1.48 mmol g^−1^ owing to the existence of nanocavities functioning as a molecular sieve. Further, microwave-assisted acid treatment was used to modify both sepiolite and palygorskite, which advances the rise in pore volume and specific surface area anticipated to Mg^2+^ leaching, especially for sepiolite. Though, the limited breakdown of these fibrous structures has not been improving the adsorption capacity of CO_2_ due to the gradual increase of the nanocavity size. Following, the amine modification of both fibrous clay minerals was performed by applying distinct techniques such as impregnation with PEI, grafting with APTES, and double functionalization by grafting with APTES and then impregnation with PEI. In each scenario, it has been noticed that the amine species functionalization supports the chemical interaction between CO_2_ molecules and the amine species. While it also creates the difficulty of the nanochannels, adsorption holds position mostly on the external surface of the fibers. Lastly, the combination of amine species by double functionalization showed an improvement in CO_2_ uptake as 2.07 mmol g^−1^ at 1 atm pressure and 338 K allocated to a higher quantity of accessible amine sites aside from higher uptake temperature, that preferred the diffusion of CO_2_ molecules into the adsorbent. Similarly, Jing *et al.*^135^ used a string of acid-treated sepiolite-supported PEI solid adsorbents with different loading. Well-dispersed Sep/PEI fibers were enclosed by PEI with a thickness of about 10 nm, and these fibers lay close to each other to produce a multi-layer cage-like structure with plentiful space for CO_2_. At 50 wt% loading of PEI, the uptake of up to 2.48 mmol g^−1^ at 348 K was achieved in mixed gases comprising 60 vol% CO_2_. Irani *et al.*^136^ synthesized an inorganic–organic CO_2_ sorbent by functionalizing TEPA onto acid-altered nanosepiolite with intended CO_2_ adsorption phenomena as shown in Fig. 13, with achieved uptake of 3.8 mmol g^−1^ for 1 vol% CO_2_ in N_2_ alongside ∼1 vol% H_2_O at 333 K. TEPA comprises two varieties of amine groups: primary (R_1_–NH_2_), and secondary (R_1_–NH–R_2_) amines. The stoichiometry of the reaction reveals the high capacity of these two varieties of amines as about 0.5 mol and 1.0 mol of CO_2_ per mole of amine in dry and humid conditions, correspondingly.

Wang *et al.*^137^ used kaolinite and montmorillonite natural clay minerals as supporting adsorbents, pre-modified by acid- or alkaline-treatment to enhance their textural properties, *i.e.*, surface area and pore volume, for adapting the CO_2_-philic PEI. Among them, the montmorillonite modified by 6 M HCl (Mon-HCl-6M) presented improved porosity with a surface area of 253 m^2^ g^−1^ from 72 m^2^ g^−1^, and pore volume of 0.71 cm^3^ g^−1^ from 0.16 cm^3^ g^−1^. Next, the modification with PEI (50 wt%) on Mon-HCl-6M, the CO_2_ uptake reached 112 mg g^−1^ at 348 K under dry conditions. This has been further improved by moisture addition (*ca.* 3 vol%) to get 142 mg g^−1^, owing to the variation in the interaction mechanism between the amine and CO_2_ in the existence of moisture. Pozuelo *et al.*^128^ utilized a large-variety of low-cost clay minerals to evaluate their performance as support of amine-containing adsorbents for CO_2_ uptake. Bentonite, montmorillonite, sepiolite, palygorskite, and saponite were hydrated and modified in three ways: (a) grafting with AP and DT organosilanes, (b) impregnation with PEI, and (c) double functionalization by impregnating previously grafted samples. Under dry circumstances, at 1 bar pressure and 318 K, grafted and impregnated samples of sepiolite-DT and palygorskite-PEI generated as high as 61.3 and 67.1 mg g^−1^ of CO_2_ adsorption capacities, respectively. However, double-functionalized samples experienced pore-blocking expected the high organic loading and displayed lower CO_2_ uptake than those attained by specific impregnation or grafting. The presence of 5% H_2_O in the feed gas resulted in an increment of CO_2_ uptake from 17 to 27%.

**Fig. 14:**
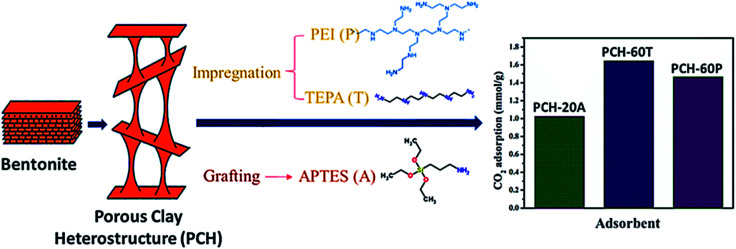
The CO_2_ adsorption capacity of amine-functionalized PCH, *via* grafting APTES and *via* impregnation with PEI or TEPA [data from ref. 138].

Porous clay heterostructures (PCHs) are inorganic structures with high versatility for a wide range of applications. The substitution of the cation given to counterpoise the negative charge of the smectite layer by a bulkier one permits the preparation of these structures with modular porosity with high physical stability. Generally, the high microporosity of PCHs permits its help in the separation and adsorption of CO_2_ or small hydrocarbons.^127^ Recently, Vilarrasa *et al.*^138^ prepared porous clay heterostructures from bentonite, then modified with amine species, *via* grafting by APTES and impregnation by PEI or TEPA. The findings revealed a CO_2_ adsorption capacity of 1.023 mmol g^−1^ with APTES grafting, and 1.644 and 1.465 mmol g^−1^ with TEPA and PEI, respectively, as can be seen in Fig. 14.

### 2.5. Porous carbon-based materials

**Fig. 15:**
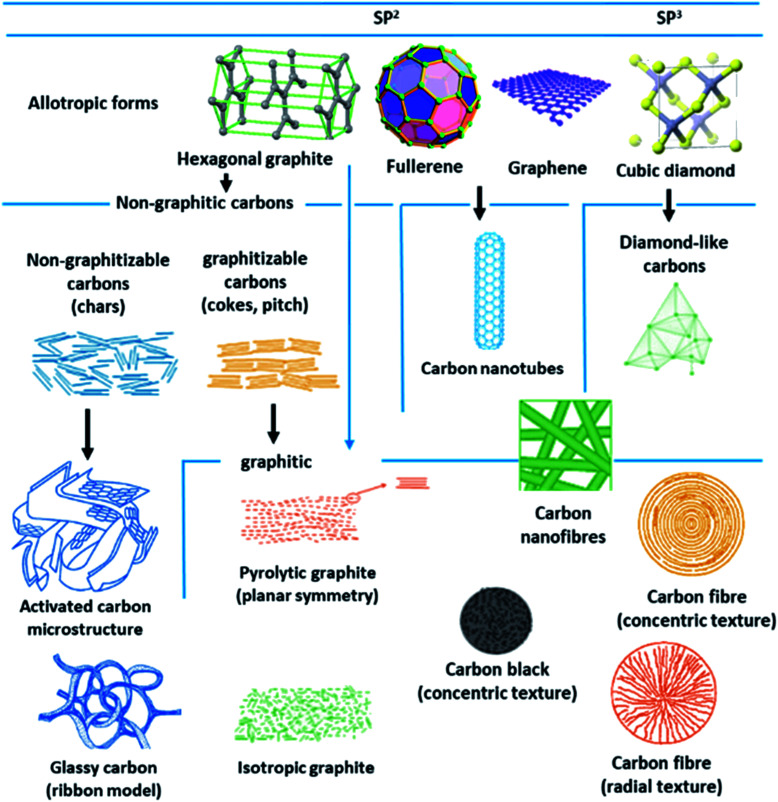
Different structures and allotropes of carbon (reproduced by permission from ref. 141 Copyright 2006, Elsevier).

Porous carbon-based adsorbents are progressively gaining interest in CO_2_ capture and are desirable owing to their huge accessibility, physiochemical stability, affordability, and flexibility to tune their porosity.^139,140^ Most of the carbons are present in the allotropic form of graphite, although fullerenes and their derivatives, diamond-like carbons organizing a shorter collection of carbon forms. Depending on the degree of crystallographic order in the third direction, allotropic types of graphite can be categorized into graphitic carbons and non-graphitic carbons. Further non-graphitic carbons have been divided into graphitizable and non-graphitizable carbons. Stepping up from nanoscale to micro-scale, carbons display extremely distinct structures. The granular and powder-activated carbons are conventional carbon adsorbents. New types of carbon are also employed as potential sorbent like fabrics, activated carbon fibers, and felts can be synthesized using different types of precursors covering coal, rayon, petroleum pitch, or viscose. These materials have a porous carbon structure, which comprises small quantities of various heteroatoms, such as hydrogen and oxygen. The existence or absence of surface groups shaped by heteroatoms that bond themselves to the carbon atoms at the edges of the basal planes provide an escalation to carbons with diverse chemical properties. The physicochemical properties are important for the behavior of carbon adsorbents.^141–144^Fig. 15 illustrates some of these carbon structures and their forms.

Activated carbons for CO_2_ adsorption are made through carbonization and physical/chemical activation of a wide range of natural sources (biomass,^145^ glucose,^146^ plant-based,^147^*etc.*) and synthetic precursors (triazine-based POPs,^148,149^ petroleum pitch,^150^*etc.*). Based on their surface characteristics and preparation technique, activated carbons are classified into powdered activated carbon, granulated activated carbon, spherical activated carbon, impregnated carbon, and polymer-coated carbon. Sevilla *et al.*^151^ used viable porous carbons synthesized by hydrothermally carbonized biomass (sawdust) and polysaccharides (cellulose and starch) with chemical activation (KOH/precursor = 2) at 873 K, named as AS-2-600. It delivered a higher capacity of CO_2_ uptake as 4.8 mmol g^−1^ at 298 K. This remarkable capacity to adsorb CO_2_ is principally anticipated due to the existence of 0.8 nm narrow micropores, while the BET surface area of 1260 m^2^ g^−1^ performs a less important role.

Attempts to manipulate carbon structures are mainly applicable to the molecular sieving carbons (MSC) or carbon molecular sieves. MSC is the porous carbon skeletal framework that persists following the pyrolysis of a polymeric precursor. MSCs porosity involves a high BET surface area of up to 4000 m^2^ g^−1^ and nanometer size uniform pores. Generally, MSCs deliver better relative adsorptive strength associated with spherical graphitized polymer carbon and graphitized carbon black sorbents. Wahby *et al.*^152^ used C-1012 with 2000 m^2^ g^−1^ of surface area and its CO_2_ adsorption capacities at 1 bar were 232, 132, and 79.3 mg g^−1^ at temperatures of 273, 298, and 323 K, respectively. They suggested that the existence of a fine-tuned microporosity, aside from a great volume of narrow micropores significantly improves the amount of CO_2_ capture. Furthermore, these tiny micropores appear to be the main reason, heading to get full CO_2_ adsorption capacity, even while achieving adsorption at a temperature similar to the anthropogenic releases of CO_2_.

When comparing carbons with zeolites or MOFs, these are less polar and consequently give weak CO_2_ adsorption affinity. Their typical downside can be surmounted by initiating heteroatoms (very frequently nitrogen, occasionally other elements) incorporated into the frameworks of carbon or applying surface functional groups. The goal was to improve the capacity of CO_2_ uptake and affinity towards its selectivity by instituting simple nitrogen-functionalities into frameworks of porous carbons. The higher N content in AC will increase its adsorption capacity. This process has been utilized to imitate the amine scrubbing procedure and enhance the hydrogen-bonding interactions among CO_2_ molecules and C–H groups, leading to a higher CO_2_ adsorption capacity. Besides, CO_2_ gas is acidic in nature and is likely to be adsorbed on the basic groups such as N-species. Additionally, doping N atoms into activated carbons by incorporating on its surface, which is quite stable in contrast with the oxygenated functional groups such as COOH, OH, and CO, *etc.* Though more content of N does not promise the improvement in the capacity of CO_2_ capture, moreover this is associated with the nitrogen species type used and their structure on the surface of activated carbon.^153^ When comparing with other N-containing species like pyrrole-like N species, pyridine and pyridine *N*-oxide, pyridine-like N species significantly enhances the capacity of CO_2_ uptake, due to their stronger basic nature.^154^ Usually, the N atom can be incorporated on the carbon surface in two ways. (i) Physical mixing as N atom impregnation on the carbon surface. For instance, the porous structure of carbon can impregnate with melamine, aniline, acetonitrile, and PAN.^155^ (ii) Grafting or doping by amine functional species from chemical compounds or reagents like ammonia, urea, urea–formaldehyde (UF), and melamine–formaldehyde (MF)^156^ to activate the carbon surface; consequently, a new bond is developed because of chemical reactions. The first one was studied largely; however, it undergoes several challenges due to blockage of a pore in minor pore volumes, which limits the loading of N content and boosts the volatilization of compounds holding N with rising temperature. Hence, grafted molecules show greater stability.^157^ Still, the impregnation technique is generally favored owing to its ease of preparation, cost-effectiveness, and it offers better chemical-loading capacity.

Likewise, higher CO_2_ uptake and improved selectivity have been achieved by doping a small amount of S atoms, largely as oxidized-S. The heteroatom doping is found to stimulate microstructure tuning with a very much organized framework containing a fine pore network, high surface area, and high sp^2^–C ratio. The established phenomenon of the variable pore framework involves hydrogen-bond connections. N,S co-doped honeycomb carbon displays a comparable CO_2_ uptake of 4.7 mmol g^−1^ at 273 K, to N-doped pillaring-layered carbon (NC) with 7.3% of N content.^158^ Alkali cation functionalization on carbon pores can advance the high basicity and polarizing ability of materials and consequently improve CO_2_ adsorption. Chen *et al.*^159^ used N-doped porous carbon attained from coconut shell by modification with urea, and KOH activation (NC-650-3) and demonstrate CO_2_ capture of 5 mmol g^−1^ at 298 K and over 7 mmol g^−1^ at 273 K, for 1 bar pressure. This greater adsorption has been credited to its high content of nitrogen and microporosity.

**Fig. 16:**
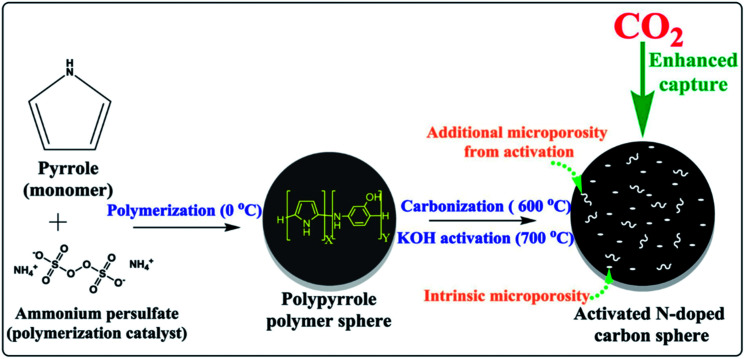
Illustration of the synthesis of activated polypyrrole-derived carbon spheres (ACS-4-6-2) (reproduced by permission from ref. 162 Copyright 2018, Elsevier).

Nandi *et al.*^160^ used highly N-doped activated porous carbon monoliths (ACMs) with a surface area of 2501 m^2^ g^−1^ containing 1.8 wt% N, which were prepared by carbonization and physical activation of mesoporous polyacrylonitrile (PAN) monoliths for CO_2_ capture. These ACMs displayed enhanced CO_2_ capture of 5.14 mmol g^−1^, 11.51 mmol g^−1^ at 298 K and 273 K, respectively, under ambient pressure. Compared to many porous carbon-based adsorbents, Ma *et al.*^161^ used SA-2N–P with BET 1740 m^2^ g^−1^ and its nitrogen content 3.38% for CO_2_ capture showed better capacity as 4.57 mmol g^−1^ under ambiance and 8.99 mmol g^−1^ at 273 K. This can be explained as pyrrolic nitrogen that usually has significantly more influence on CO_2_ adsorption than pyridinic nitrogen. Similarly, Dassanayake *et al.*^162^ used ACS-4-6-2 with a surface area of 1079 m^2^ g^−1^ alongside 3.7 wt% nitrogen content resulting in CO_2_ uptake of 5.42 mmol g^−1^ at 298 K, 7.73 mmol g^−1^ at 273 K at 1 bar pressure. This enhanced CO_2_ capture was attained by regulated activation of polypyrrole carbon spheres with immediate maintenance of spherical morphology and a great increment in ultra-microporosity. A schematic preparation process is shown in Fig. 16.

Waralee *et al.*^163^ used nitrogen- and oxygen-enriched activated carbons, prepared *via* urea incorporation, air oxidation, and KOH activation. At 298 K, the high N-content samples demonstrated an improved uptake in the pores at moderate pressures (*i.e.*, 1 bar) and affinity at low pressures. While at 273 K, it still showed greater affinity; however, its adsorption capacity was slightly lowered. Subsequently, a Grand Canonical Monte Carlo simulation was presented macroscopically and microscopically to examine the performance of carbon dioxide uptake. The experiments were performed by using the graphitic slit pore (pore width: 0.7–1.5 nm) for pristine, and by applying pyridine (N-6), and hydroxyl (OH) functional groups on it. (i) Adsorption affinity: the active site of the surface functional group is prominent in which carbon dioxide has a strong affinity towards N-containing species for all examined temperatures. The observed findings of simulated results were reliable with investigated data. (ii) Adsorption capacity: the effect of pore width has shown a high significance. Nevertheless, efficient pore widths fluctuated with temperature. More adsorption uptake in the pores with appropriate width followed, as did the correlation between the energy of motion and the packing of adsorbed molecules in a sort of optimizing the energy. The energy of motion is highly influenced at higher temperatures, while the corresponding packing is vital at the lower values.

A novel kind of extremely hierarchical porous carbon (HPC) along with a huge increment of BET surface area up to 2734 m^2^ g^−1^ and improved total pore volumes up to 5.53 cm^3^ g^−1^ were prepared through customized carbonization of several metal–organic frameworks. HPCs are extremely sp^2^-bonded graphitic in nature with a great percentage of defective carbon structures. In most studies, the adsorption capacity of CO_2_ capture in HPCs is greater than that in their MOF counterparts. At high-pressures, 30 bars and 298 K, the adsorption capacity of HPCs for CO_2_ uptake was more than 27 mmol g^−1^. It appears to be a straightforward correlation between the CO_2_ uptake and the surface area. For every 1000 m^2^ g^−1^ increase in surface area, there is an increase in CO_2_ adsorption capacity by 10 mmol g^−1^ at 30 bar and 300 K. Owing to their physicochemical stability and improved performance of CO_2_ uptake, these adsorbents are favored over their counterpart MOFs in PSA/VSA applications.^164^

**Fig. 17:**
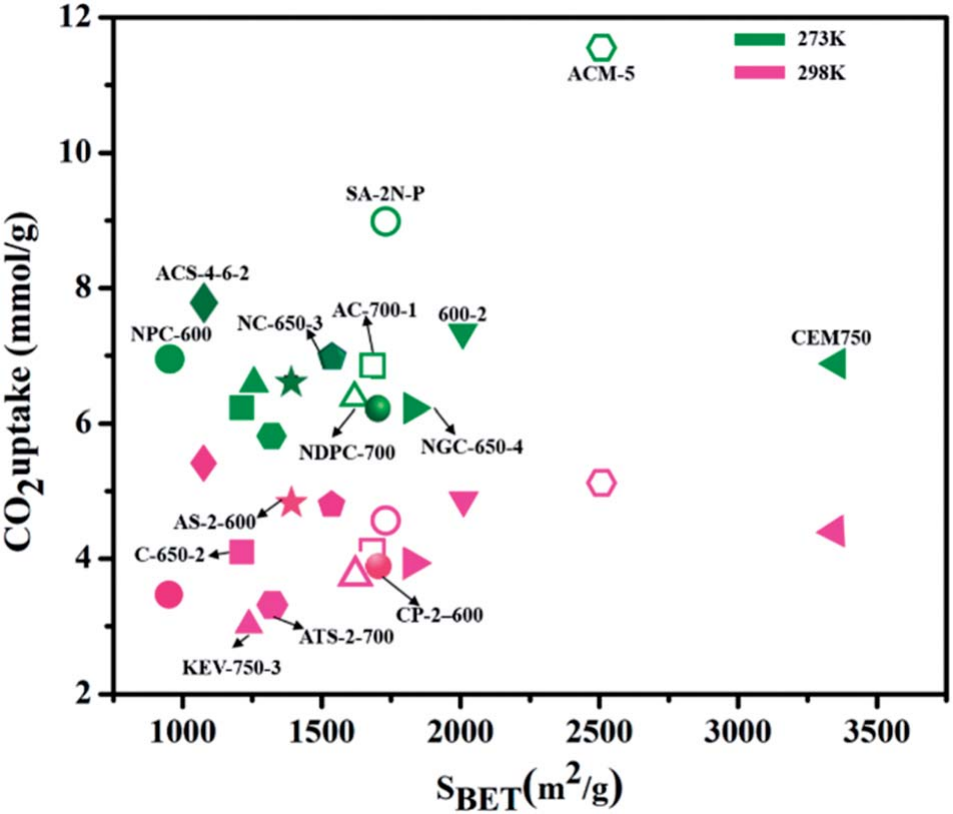
The literature of different porous carbon-based adsorbents for CO_2_ capture at different temperatures (273 K, 298 K) for 1 bar pressure w.r.t. surface area (some have been converted to these units from the originally reported units).

In recent years, various other nanocarbon forms (CNTs, graphene, and GO) of distinctive surface morphology have drawn interest as CO_2_ adsorbents. Wang *et al.*^165^ used hierarchical N-doped carbon nanotubes (NCTs) with well-regulated aspect ratios for CO_2_ capture. These are prepared by coating distinct loadings of 3-aminophenol/formaldehyde resin (APF) on the exterior layer of silica nanotubes, and carbonizing in N_2_ at 700 °C, then eliminating the silica and Ni template by hydrofluoric acid etching followed by K_2_CO_3_ activation. The achieved ANCTs showed pore volume and micro-surface area of 0.45 cm^3^ g^−1^ and 1195 m^2^ g^−1^, respectively, and the subsequent uptake increased by 50% as 4.50 mmol g^−1^ at 273 K and 1 bar. Mishra *et al.*^166^ prepared large-scale, low-cost, graphene nanosheets by using hydrogen exfoliation, with enhanced CO_2_ capture of 21.6 mmol g^−1^ detected at 298 K and 11 bar pressure, as related to other nanocarbon forms, indicating their potential as CO_2_ adsorbent material for industrial applications. Fig. 17 illustrates the literature of various carbon-based adsorbents along with their surface area used for CO_2_ uptake.^167–176^

### 2.6. Polymer-based adsorbents

**Fig. 18:**
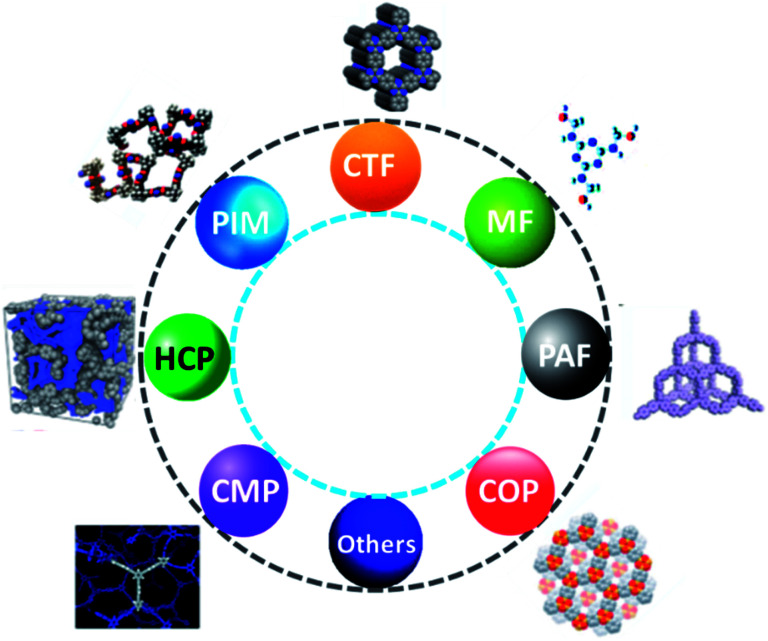
Chemical structures of diversified porous materials.

Recently, sequestration of CO_2_ in solid adsorbents through the physisorption and their effective conversion into values added sufficient chemicals are significant prior areas of research. However, the innovation of effective solid CO_2_ adsorbents together with their regeneration efficiency, chemical and mechanical stability are the most challenging objectives. In this context, porous organic polymers (POPs) owing to their lightweight, chemical stability, and high specific surface area, structural diversity including topologies and chemical functionalities, and porosity at the nanoscale level are highly advantageous. Moreover, POPs have vast potential in various applications such as sensing, gas storage and separation, optoelectronics, energy storage, and catalysis due to their lightweight and composition (elements like H, C, N, and O are strongly connected by covalent bonds).^177^ Besides, the most important feature of POPs is taking the CO_2_ gas in a particular ambient condition in the reversible adsorption process. Until now, various forms of POPs have been studied to capture CO_2_ as hyper cross-linked polymers (HCPs), polymer with porous aromatic frameworks (PFA), porous melamine–formaldehyde (MF), covalent organic polymers (COP), polymers with intrinsic microporosity (PIMs), conjugated microporous polymers (CMPs) and polymer with covalent triazine based framework (CTF),^178^ as displayed in Fig. 18.

Over the years, a wide range of POP materials is obtained with many polycondensation reactions like Schiff-base condensation, Friedel–Craft reactions, free-radical polymerization, Diazo-coupling, thermal/solvothermal condensations, cross-coupling reactions, metal-catalyzed C–C homo reactions, and thermal/chemical switch reactions for suitable applications due to their high surface area and porous nature.^179^ More importantly, CO_2_ uptake capability is mainly dependent on the surface area, as the CO_2_ adsorbent uptake rises with the increase in the uptake pressure. This represents that tuning the surface property could be advantageous for CO_2_ uptake capability.

An MF-porous melamine–formaldehyde amino resin with the content of hydroxymethyl groups was obtained by the polycondensation of additional formaldehyde with melamine. Moreover, the Mannich reaction occurs during the grafting of polyethyleneimine (PEI) onto the porous amino resin and continues to form (MF-*g*-PEI). The stability of MF-*g*-PEI adsorbent is enhanced with the help of excessive formaldehyde, utilized in the polycondensation, which will provide more active sites to be associated with PEI. In the novel method of direct grafting, the solid amine adsorbent into the PEI without the presence of an intermediate provides enhanced adsorption kinetics and generation performance with better features (pore volume of 0.76 cm^3^ g^−1^ and surface area (BET) of 460 m^2^ g^−1^). At 283 K, the MF-*g*-PEI showed a CO_2_ uptake of 1.32 mmol g^−1^ with 48.9% amine consumption efficiency, along with 11.35% of PEI loading. PMFRs-porous melamine–formaldehyde resins are one of the most recently studied materials for CO_2_ adsorption/separation applications, because of their high surface area, high binding affinity with CO_2_, good stability, and large pore volume. Most PMFRs are prepared from melamine due to their weak basicity, low cost, and has abundant nitrogen (N). However, due to instability issues, PMFRs prefer physisorption rather than chemisorption of CO_2_.^180^

Recently, microporous polymers with nanopore size, also known as hyper cross-linked polymers (HCPs), are showing significant interest due to their diversiform aromatic monomers, simple Friedel–Crafts reaction, and high yields. In HCPs, the well-swollen chains with plentiful rigid cross-linking bridges, are produced by the Friedel–Crafts reaction.^181^ Davankov-type resins are the most studied, which are made from polystyrene and have an upper surface area of about 2000 m^2^ g^−1^. Wang *et al.*^182^ reported the CO_2_ uptake of 99.0 mg g^−1^ at 273 K, and 55.2 mg g^−1^ at 298 K for 1.0 bar pressure, respectively, with the help of (HCP) microspheres with a hollow core–shell structure prepared by a hard template method, and the results show that HCPs-5% represents an important role of ultra-micropores in the adsorption process. Fang *et al.*^183^ described the synthesis of Friedel–Crafts reaction-based HCPs from 9-phenylcarbazole. The as-synthesized HCPs showed the CO_2_ uptake capacity (at 273 K and 1 bar pressure) of 10.4 wt% with a specific surface area up to 769 m^2^ g^−1^. Hou *et al.*^184^ reported an excellent CO_2_ uptake of 5.63 mmol g^−1^ at 273 K temperature, and 1 bar using high surface area HCPs with different polycyclic aromatic hydrocarbons. Penchah *et al.*^185^ proposed thermodynamic modeling for CO_2_ adsorption using benzene-based HCPs. For the industrial application of benzene-based HCP, synthesis was carried out at the optimal time, temperature and pressure of 13.6 h, 294 K, and 7.8 bar, respectively.

Another type of POPs is CMPs- (conjugated microporous polymers), generally obtained from aromatic building blocks. CMPs originate from different routes such as permanent micropores, conjugated organic frameworks, and π-electron conjugated systems.^186,187^ To enhance the performance of CMPs towards CO_2_ adsorption, researchers introduced electron-rich groups such as N, O, Si, and other heteroatoms. Xu *et al.*^188^ prepared CMPs based on 1,4,5,8-naphthalene tetracarboxylic dianhydride (NTDA), tetrakis (4-aminophenyl) ethene (TPE-NH_2_) core with pyromellitic dianhydride (PMDA), and 3,3′,4,4′-biphenyl tetracarboxylic dianhydride (BTDA). Here, the tetraphenylethene core and anhydride linkers improve p-conjugation, rigidity, and planarity contributing towards CO_2_ uptake. Moreover, these polymers are excellent adsorbents for CO_2_ gas storage and separation. The CO_2_ adsorption uptake of CMP@1, 2, and 3 at temperature and pressure of 273 K and1.05 bar reached 2.27, 1.56, and 1.6 mmol g^−1^, respectively. Similarly, Zhou *et al.* stated^186^ that the uptake capacity of CO_2_ adsorption of CMPs depends upon the rise in pressure continually and reached 12.70, 11.55, and 10.23 wt% at 273 K, and 1.0 bar. However, the CO_2_ adsorption uptake depended on the micropore-specific area, which was in the order of CMP1 > CMP2 > CMP3. This explains that high adsorption uptake can be cationic cyclization polymerization generated with high microporosity of polymers.

Polymers with intrinsic microporosity (PIMs) are considered to have low surface areas and high free volumes with a continuous interconnected network of intramolecular voids. PIMs generate porosity from the rigid and contorted macromolecular chains that are not packed inefficiently.^189^ PIM polymers show fused-ring sequences, which make PIMS restrict free rotation and contortion ladder-type assemblies turn to avoid the packing efficiency of polymers. Sekizkardes *et al.*^190^ reported very high CO_2_/N_2_ selectivity in the presence of dry and humid conditions for post-combustion CO_2_ capture. PIMs are obtained through an acid–base interaction (chemisorption) with alkylamine incorporation. The amine-appended PIMs presented a nearly four-fold improvement in CO_2_ loading uptake, which is 1.6 mmol g^−1^ at temperature and pressure of 298 K and 0.15 bar compared to the pristine material.

Porous aromatic frameworks (PAFs) are among the most studied and important POPs due to their high surface area and tremendous physicochemical stability. Normally, PAFs are synthesized by using irreversible cross-coupling reactions and composed of phenyl-ring derived fragments with different functional groups by synthetic chemistry routes. PAFs consist of 2D/3D periodic aromatic frameworks, which are obtained by the effective assembly of organic building blocks through covalent coupling reactions.^191^ PAFs can be used for TSA (temperature swing adsorption), VSA (vacuum swing adsorption), and PSA (pressure swing adsorption) to increase the CO_2_ adsorption capacity. Moreover, to improve the CO_2_ adsorption uptake, surface area, tailoring the pore size, post-modification, and heteroatom doping can be tuned. Demirocak *et al.*^192^ reported the Yamamoto coupling route to synthesize *p*-phenylenediamine based, porous aromatic framework (NPAF). The as-obtained NPAF showed a surface area of 1790 m^2^ g^−1^ with a CO_2_ uptake capacity of 3.64 mmol g^−1^ at a temperature of 273 K.

Covalent organic frameworks (COFs) are porous crystalline organic polymers that gained enormous research interest in the past 10 years. These are synthesized using organic molecules that are covalent-linkage bonded in a repeating manner to create a porous crystal that is best suitable for gas capture and storage. COPs-covalent organic polymers are another type of highly porous crystalline organic polymer.^193^ Liu *et al.*^194^ obtained a novel TPFM (bithiophene–melamine porous organic framework) prepared by a one-step Shiff-base reaction using cost-effective raw materials as S-rich thiophene formaldehyde and N-rich melamine. The raw materials show a greater surface with mesopore-controlled pore structure such as sphere-like morphology with outstanding CO_2_ uptake capacity of 3.46 mmol g^−1^ (about 16 wt%) at 273 K temperature. Xiang *et al.*^195^ reported a series of covalent organic polymers for the adsorption of various gases such as H_2_, O_2_, CH_4_, CO_2_, and N_2_. The CO_2_ uptake capacity showed at a temperature of 298 K and pressure of 18 bars was 13.5 mmol g^−1^. Furthermore, these covalent organic polymers performed better capabilities on the removal of CO_2_ from natural gas.

**Fig. 19:**
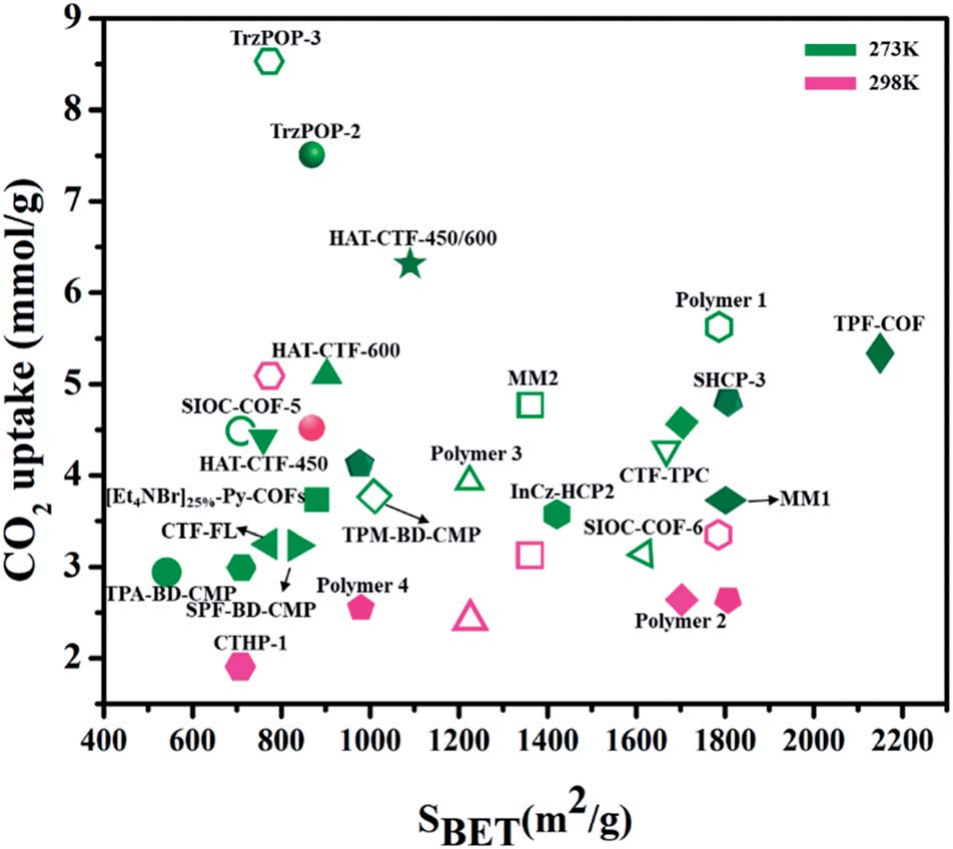
Summary of different porous POPs for CO_2_ capture at different temperatures (273 K, 298 K) for 1 bar pressure w.r.t. surface area (some have been converted to these units from the originally reported units).

CTFs – covalent triazine frameworks (CTFs) are another type of widely studied POP for CO_2_ capture. In CTFs, frameworks contain triazine pore units that simply originated from building blocks connected by covalent bonds that have advanced stability compared to many coordinative-linked materials. Liebl *et al.*^196^ reported the synthesis of chemically and thermally stable seven triazine-based porous polyimide (TPI-1 to TPI-7) polymers for CO_2_ capture. The as-obtained TPI-1 (809 m^2^ g^−1^) and TPI-2 (796 m^2^ g^−1^) showed high values of CO_2_ capability as 2.45 mmol g^−1^ at 273 K and 1 bar. Furthermore, it stated that CO_2_ uptake capabilities depend upon surface areas. Mohamed *et al.*^197^ reported the synthesis of pyrene-functionalized covalent triazine frameworks Pyrene-CTF-10 through the ionothemal treatment of 1,3,6,8-cyanopyrene (TCNPy) with (ZnCl_2_) molten zinc chloride at 500 °C and displayed good CO_2_ adsorption capacity of 2.82 and 5.10 mmol g^−1^ at 298 K and 273 K, respectively. Fig. 19 shows the different porous POPs and their surface areas for CO_2_ capture at different temperatures (273 K, 298 K) and at 1 bar pressure.^198–209^

### 2.7. Porous metal oxides

From the last decade onwards porous metal oxides (MOs) are favorable candidates for acting as CO_2_ capture materials under pre, post, and oxy-combustion conditions. These porous oxides blend with CO_2_ in the flue gas and produce carbonates. There is still a huge significance in the forthcoming, owing to their promising thermodynamic properties and ease of availability. Though, the choice of these MOs results in their CO_2_ adsorption capacity, rate of adsorption, regeneration heat, availability, thermal stability, structural and textural properties. Various porous MOs, like alkali and alkaline-earth metal oxides (MgO and CaO), together with CO_2_ molecules produce thermodynamically stable carbonates. Lately, Li, Na, and K-based silicates, or zirconates, and perovskites have also attracted huge attention as expected due to their enhanced CO_2_ capture.^210–215^

CO_2_ capture cyclic process for some porous metal oxides (MO) and metal carbonates (MCO_3_)

1 MgO(s) + CO_2_(g) ↔ MgCO_3_(g)

2 CaO(s) + CO_2_(g) ↔ CaCO_3_(g)

3 Fe_3_O_4_(s) + Fe(s) + 4CO_2_(g) → 4FeCO_3_(s)

4 Li_2_ZrO_3_(s) + CO_2_(g) ↔ ZrO_2_(s) + Li_2_CO_3_(s)

5 Li_4_SiO_4_(s) + CO_2_(g) ↔ Li_2_SiO_3_(s) + Li_2_CO_3_(s)

6 Li_5_AlO_4_ + 2CO_2_ → LiAlO_2_ + 2Li_2_CO_3_

7 Na_2_ZrO_3_(s) + CO_2_(g) ↔ ZrO_2_(s) + Na_2_CO_3_(s)

8
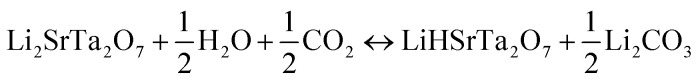

9 Ba_4_Sb_2_O_9_ + 3CO_2_ ↔ BaSb_2_O_6_ + 3BaCO_3_

10 Li_8_SiO_6_ + CO_2_ → Li_4_SiO_4_ + CO_2_ → Li_2_SiO_3_ + Li_2_CO_3_

**Fig. 20:**
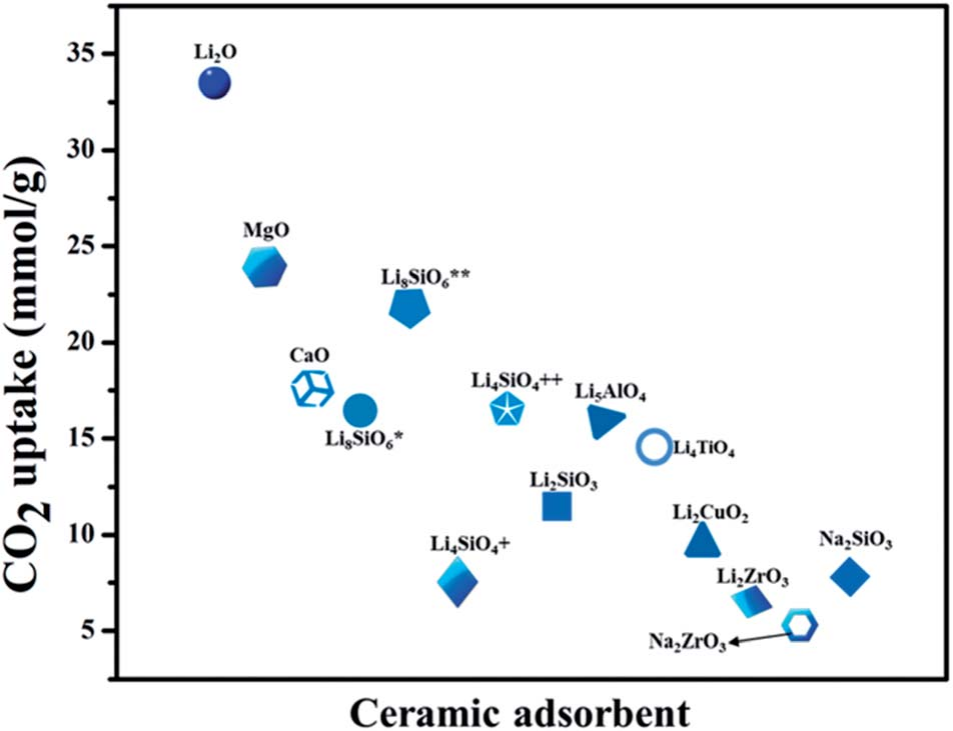
Theoretical uptake of carbon dioxide for various ceramic adsorbents (data from ref. 216).

Fig. 20 shows that the theoretical adsorption capacities of CO_2_ chemisorption for very well studied alkaline and alkaline-earth ceramics. Among most of these materials Li_2_O, MgO and CaO showed greater performance. However, Li_2_O and MgO are not considered as potential candidates for CO_2_ capture due to their reactivity and kinetics factors. In contrast, CaO is considered a promising alkaline earth-based material, along with viable commercial applications. In addition to metal oxides, lithium, or sodium phase ceramics also showed high thermal stabilities and volume changes than CaO. In Li_4_SiO_4_ (+) and Li_8_SiO_6_ (*), the highest adsorption has been relayed on the CO_2_ moles captured in each different phase as represented in eqn (10). Besides, sodium phase ceramics offered the benefit of CO_2_ adsorption in the presence of steam. Further down these materials generate NaHCO_3_ as the carbonated phase, showing twofold capacity in contrast to that of Na_2_CO_3_ under dry conditions.^216^

## 3. Conclusion

As there is a continuous day-to-day increase of anthropogenic CO_2_ in the atmosphere, due to power plants, chemical processing, overuse of fossil fuels, and deforestation, it is vital to engage in an on-going effort to reduce the consequence of global greenhouse emissions causing climate change by establishing an effective approach for capturing CO_2_. Adopting porous materials for adsorption is a promising strategy, lowering the energy necessary for regeneration related to the commercially implemented liquid amines absorption approach for post-combustion carbon uptake. In this regard, a wide range of porous adsorbents was discussed along with their modifications. MOFs are very well-arranged microcrystalline materials with a high surface area, easy to control pore sizes, and their improvement in CO_2_ selectivity is much desirable for CO_2_ capture. While the capacity of CO_2_ capture and CO_2_/N_2_ selectivity of acknowledged MOFs are not capable at low pressure. Additionally, notable problems were encountered on MOFs leading to questions on their long-term structural stability, high cost of even the most basic organic linkers, challenges in robust formulations of MOF, *etc.* Under ambient conditions, M-MOF-74 worked well in terms of CO_2_ uptake, which is mainly attributed to its Lewis acidic sites. While zeolites show promising results not far behind MOFs at low pressure shown by 13X variants and zeolites in sodium form. Silica and clays have fast adsorption kinetics, and a high capacity of CO_2_ capture under mild conditions but their present phase of advancement has limited applications. These adsorbents endure easy moisture adsorption, intense energy utilization while CO_2_ desorption (poor economic viability), and expensive pre-treatment rises the regeneration cost. Besides ZIFs show greater selectivity than MOFs for CO_2_ from other relevant flue gases. Carbon-based materials offer advantages of high stability, cheap raw materials, rapid carbonation kinetics, low desorption temperature, but possess relatively low adsorption capacities. The superior adsorption capacity was observed in activated porous carbon monoliths (ACMs) at ambient pressure. Likewise, CTF, COFs gained enormous research interest due to their high adsorption capacity. POPs give improvements in selectivity and enhanced CO_2_ uptake. Though they are limited by their high reliance on their preparation requirements, making them complex and expensive in relation to the above-mentioned adsorbents. Also, MOs exhibited especially higher uptake capabilities but necessitate high-temperature regeneration procedures. Some of the satisfactory minimum requirements for designing a solid adsorbent for CO_2_ capture are working capacity of at least 2 mmol g^−1^, >100 CO_2_ selectivity, water stability, and cost less than USD 10 per kg of an adsorbent are desirable.

There are two types of fundamental adsorption phenomena involved during CO_2_ capture. For pristine porous materials, by physisorption due to van der Waals interaction between the adsorbent and carbon dioxide molecules, including through pole–ion and pole–pole interactions among the quadruple of CO_2_ and the ionic and polar sites of the sorbent surface. At the same time, amine-modified adsorbents involve chemical reactions. Thus, it is vital to understand how the nature of amine affects the CO_2_ uptake and the rate of adsorption. The zwitterion mechanism is frequently used to describe the effect of CO_2_ and primary/secondary amines. Though, a deeper understanding of the fundamentals of CO_2_ adsorption on porous material surface may lead to the creation of far more effective CO_2_ adsorbents.

So far, the materials produced for selective capture of CO_2_ are usable, they do need substantial changes before they can be considered practical. Some fundamental issues such as physiochemical stability, performance in the presence of moisture, stability towards impurities, gas diffusion rates, reversibility and regeneration, affordability to improve economics and practical usage for porous adsorbents need to be addressed for effectively capturing CO_2_ when competing in a state-of-the-art scrubbing process. Cost and scalability will be the most significant factors from the industrial viewpoint. Any expensive material or precursors in sorbent or the processing technique is undesirable. Cost is often the explanation for promising new materials remaining in the developmental stage. Materials like MOFs and POPs could hardly be expected to compete with commercial amines in terms of cost, while they are very much regarded for their high CO_2_ adsorption capabilities. Zeolites, silica, clay, and porous carbons have potential cost-efficiency, but further developments in research and design must be made to demonstrate their full potential in sustainable CO_2_ capture in the near future. Furthermore, to meet some of these challenges, the CO_2_ adsorption can be enhanced by implementing modification approaches such as optimization of textural properties, amine functionalization, and CO_2_-philic heteroatom doping (like N, S).

## Conflicts of interest

The authors have no conflicts of interest.

## Supplementary Material
